# Experiences of mothers and significant others in accessing comprehensive healthcare in the first 1000 days of life post-conception during COVID-19 in rural Uganda

**DOI:** 10.1186/s12884-022-05212-x

**Published:** 2022-12-15

**Authors:** Mary-Grace Nakate, Sean Mackay, Eunice Ndirangu-Mugo, Valerie Fleming

**Affiliations:** 1 School of Nursing and Midwifery, Aga Khan University, Col. Muammar Gaddafi Road, P.O. Box 8842, Kampala, Uganda; 2 School of Nursing and Allied Health, Liverpool John Moores University, 79 Tithebarn St, Liverpool, L2 3ET UK; 3 School of Nursing and Midwifery, Aga Khan University, EA 3Rd Floor Sunny Plaza Wangapala Road, P.O Box 39340-00623, Nairobi, Kenya

**Keywords:** COVID-19 pandemic, First 1000 days of life, Experiences of COVID-19, Uganda

## Abstract

**Background:**

COVID-19 presented an unprecedented global public health challenge because of its rapid and relentless spread, and many countries instituted lockdowns to prevent the spread of infection. Although this strategy may have been appropriate to reduce infection, it presented unintended difficulties in rural Uganda, especially in maternal and born newborn care. For example, some services were suspended, meaning the nearest health facility was at a considerable distance. This study explored the experiences of mothers and their significant others of comprehensive care in the first 1000 days of life post-conception during the COVID-19 pandemic in Bunghokho-Mutoto sub-county, Mbale District, Uganda.

**Methods:**

A qualitative exploratory descriptive design was used with data collected in semi-structured interviews. Mothers (pregnant or with a child under 2 years) and their significant others were purposively recruited for this study. The sample size (*N* = 14) was determined by data saturation. Data. were analysed using thematic analysis.

**Results:**

One theme emerged “Increasing barriers to healthcare”, which encompassed six sub-themes: accessing healthcare, distressing situations, living in fear, making forced choices, navigating the gatekeepers, and ‘coping with increased poverty.

**Conclusion:**

This study found that the COVID-19 pandemic increased barriers to accessing healthcare services in the region. Participants’ narratives emphasised the lack of access to expert care and the shortage of skilled health workers, especially midwives.

**Supplementary Information:**

The online version contains supplementary material available at 10.1186/s12884-022-05212-x.

## Background

A pneumonia of unknown cause was first announced in Wuhan, China in 2019 and subsequently, the disease was named coronavirus disease 2019 (COVID- 19) [1]. COVID-19 spread to many countries across the world with the World Health Organization officially announcing the coronavirus (COVID-19) pandemic on 11 March 2020 [1]. Meanwhile in Uganda, the first case of COVID-19 was confirmed on March 21, 2020**,** and a countrywide lockdown was declared on 22^nd^ March 2020 [2]. Uganda used its experience with cholera to implement an immediate and strict lockdown one day after the first case was announced [2, 3]. To prevent the further spread of the virus, the movement of people by private vehicles was prohibited from 22:00 (local time). In addition, a curfew from 19:00 to 06:30 was put in place on Tuesday, March 31. Thereafter, all members of the public, except for individuals transporting cargo, were instructed to stay indoors. Gatherings of more than five people were banned as a precautionary measure. Shops selling non-food materials were closed and a ban on all public transport remained in place. Schools and institutions of higher learning, bars, and cinemas were closed indefinitely. Public and private transport was restricted, and people were only allowed to move with permission from the local government or resident district commissioners until May 2020 marking the first lockdown [2–6].

During the first lockdown, when the pandemic began to take hold locally, the number of reported cases of COVID-19 was initially low in Uganda compared with other countries. The number of reported cases of COVID-19 initially remained low at 80 between March and June 2020. However, when the lockdown was removed, there was a spontaneous increase in COVID-19 cases between June and August from 80 to 739 cases [6]. Because of the complete lockdown, non-essential visits to health facilities like antenatal care (ANC) services and childhood immunisation clinics, were prohibited for a short time; from 23 March 2020 to 21 April 2020 [7]. During this complete lockdown, laboring mothers were advised to contact the local leaders in the urban communities for guidance regarding accessing the ambulances to take them to a healthcare facility. Similarly, in rural communities, mothers were encouraged to access the health facilities using the available means of transport as long as they sought permission from the LC Chairperson. The mothers and their significant others encountered challenges using the available means of transport as they were mistakenly harassed during the restrictive law enforcement time as they struggled to reach the health facilities [8].

This process delayed mothers in accessing the services in time. Similarly, due to the restrictions on transport, health workers especially in the rural areas took time to travel to their workplaces in the rural communities unlike their counterparts in the urban area. Finally, the first lockdown was eased on 26^th^ May 2020 with restrictions: private cars with only three people and general merchandise shops allowed with strict social distancing [1].

The first 1000 days of life extend through a woman’s first day of pregnancy to her child’s second birthday [9]. During this time, the child’s brain and other vital body parts begin to grow and develop rapidly. Therefore, this period offers a unique opportunity to consider and implement interventions that aid the child’s developmental processes [10]. The child’s parents and significant others in the family are key role players that must be targeted to ensure the child’s well-being and healthy development during these first 1000 days of life. This period has been the target of many initiatives in the 30 years since the Safe Motherhood Initiative was launched by United Nations agencies to reduce the number of maternal and neonatal deaths [11]. The government of Uganda set activities at all the health facilities, both urban and rural, that stop preventable maternal deaths by ensuring mothers can access care at every stage before, during, and after their pregnancy. The mothers receive this information during the four least minimally recommended antenatal care visits, at which they are given information related to the prevention of the transmission of HIV from mother to child HIV-positive [12, 13]. Research shows that Uganda had not yet achieved the best attendance of antenatal and postnatal care services both in the urban 65% and rural settings 58%, and the facility birth is at 62% [13–15]. To mitigate the effect of nonattendance of the ANC, the government of Uganda has trained a team of individuals at the lower level and their work is to encourage communities to access the available health services and to provide the basic needs in health. The Village Health Teams (VHT) play a significant role in ensuring that mothers reach the health facilities and receive the available services, especially in rural communities. The restrictive measures negatively affected the already strained situation in Uganda when the mothers and their significant others could not access the health facilities to prevent maternal and child death. The VHT was not able to access the community members or offer Home-Based Care in fear of transmission contraction of COVID-19 In addition, the health workers were finding it hard to reach the health facilities in time because of the challenges in public transport, especially in the urban setting. On the other hand, the rural setting may house their midwives near the health facilities, but the mothers were not able to reach the health facilities in time [7]. To overcome the challenge of bureaucracy in obtaining permission to travel and the inability to access health services in time with this situation, some of the mothers and their significant others chose to seek health services from the Traditional Birth Attendants (TBA) who were near the mothers and were willing to offer the maternal services including deliveries [16]. World Health Organization [WHO] (2020) states that “qualified human resource is regarded as a key number one component for a successful health system”, this situation negatively affected the mothers who can access care at every stage before, during, and after their pregnancy and childcare outcomes during this time.

Significant others refer to the members of the family that make decisions regarding the well-being of the pregnant mothers, newborn babies, and infants in this family. They may include the head of the family, the woman’s or parents-in-law, or her parents. These individuals may be significant to mothers who are not employed or depend entirely on others for survival and decision-making [17]. This might not hold among mothers who are empowered and are able to make decisions on issues related to their health, their newborn babies, and infants. In the Ugandan context, mothers, especially in rural communities may not be fully empowered to make health decisions, and they may depend on the decisions made by significant others in their family for support and decision-making on health issues [18]. The perceptions and experiences of these significant others may, therefore, affect the outcomes of pregnancy, newborns, and infants.

The government of Uganda aims to provide required maternal and child healthcare including antenatal, intrapartum, newborn care, and infant care at all health centres (HC), although services are limited at lower-level health facilities (e.g., HCII). Communities, especially mothers and their families, are encouraged to access all required healthcare services, including maternal and child healthcare services, to ensure comprehensive care is received throughout the first 1000 days of life [10]. However, the COVID-19 lockdown in Uganda meant that the availability of professional care during this period was limited [11]. Research shows that many mothers and their significant others had limited access to health services like ANC, postnatal care, family planning services, HIV health services like refills, and young child clinics that provide immunisation services, due to the restrictive procedure and poverty, especially, among the rural communities if the mothers and their babies accessed the health facilities, there were delays at the health facilities due to the limited numbers of health workers because they experienced challenges in reaching their workplaces because of the limited availability of transport services and supply of Personal Protective Equipment (PPE) [16, 19].

On the other hand, the increasing number of patients at rural health facilities during the pandemic also meant that maternity services were at high risk of failing when some (already understaffed) facilities were turned into COVID-19 care units [19]. In addition, a distance less than two metres from a potentially infected patient were thought to pose a major risk for healthcare workers [20]. However, staff and patients in maternity services cannot adhere to these social distancing restrictions as the field involves close patient contact. A previous study noted that ‘the inadequate availability of PPE has increased fear among midwives continuing to provide care for mothers, putting themselves and their families at greatest risk. Likewise, pregnant mothers fear contracting COVID-19 at healthcare facilities, particularly in rural areas [21]. This led to mothers seeking help too late, resulting in increased stillbirths, neonatal deaths, and maternal deaths, thereby increasing Uganda’s already high mortality rates [22].

The experience of lockdown/isolation for mothers and their significant others in the family also had the potential to have serious ramifications for the socio-cultural structure of society, particularly access to comprehensive healthcare [23]. Therefore, this study aimed to describe the experiences of mothers and their significant others in accessing comprehensive healthcare in the first 1000 days of life post-conception during COVID-19 in rural Uganda.

## Method

This research was conducted using a descriptive qualitative design. This design allowed us to conduct in-depth interviews with mothers and their significant others in the family The participants narrated stories of their experiences of accessing comprehensive healthcare. during the COVID-19 pandemic. The design guaranteed the richness of data which allowed us to gain a deeper understanding of their experiences individually [24].

### Setting

Mbale District is in the Eastern region of Uganda. It is bordered by Sironko and Bulambuli districts in the North, Bukedea district in the Northwest, Budaka, and Pallisa in the west, Tororo and Butaleja districts in the south-west, Manafwa, and Bududa districts in the east. It lies between latitudes 00,450 North and longitudes 340 East and 350 East. The district has a total area of 538.16 sq. km and a population density of 915 persons per square km. This district has three constituencies which include Mbale municipality, Bungokho North, and Bungokho South [25]. Secondly, it has a total population of 465,000 people across its 27 sub-counties. Its central town and its commercial center are Mbale Municipality, with a population of 96,189 people [26]. In 2015 Mbale district had an outbreak of Cholera registering 143 cases and eight deaths. During that time, the government of Uganda instituted restrictive measures and the outbreak was well-managed. This time, during the COVID-19 pandemic, statistics show that Mbale Regional Referral Hospital, the reference point for those who had COVID-19 in the Mbale district registered 847 cases, discharged 669, and had 149 death [25]. The researchers used this history of managing infectious diseases to select this district. The study was conducted in the sub-county of Bungokho-Mutoto, which has five parishes and 82 villages, out of which four villages namely, Luyekhe B, Bukasakya, Bunamwani, and Makere were selected.

### Sampling design and sample determination

BungokhoMutoto was randomly selected from the six high-index sub-counties in the district namely Nakaloke, Namabasa, Namanyonyi, Bukasakya, Busiu, Bungokho, Bungokho-Mutoto, and Industrial division. These villages were numbered, papers were shaffled and the researchers randomly picked one that indicated Bungokho-Mutot. With the help of the LC 1 Chairperson and the VHT, the researchers conveniently selected the villages that were on the main road and had easy access to the health facilities in this sub-county. Secondly, this area was sampled because it had an activity; “Mothers Heart Uganda Mutoto Mbale” that aimed at promoting safe motherhood had been initiated in this sub-county, therefore, this community had the advantage of receiving services that would promote maternal and child healthcare outcomes [27].

Similarly, information-rich mothers in the 1000 days of life and their significant others were purposively selected to participate in the study. The researchers interviewed the participants at times convenient to them until repetitive statements were received from the subsequent interviews. This was the point at which data saturation was reached with a sample size of 14 participants [28].

### Study procedure and data collection

To gain access to the participants, the researchers approached the Local Council (LC) II Chairperson of the Bungokho-Mutoto sub-county with a permission letter from the Uganda National Council for Science and Technology who eventually, introduced them to the LC I chairperson of the Bukasakya parish Bungokho-Mutoto sub-county. The LC1 Chairperson introduced the researchers to one of the Village Health Team (VHT) members for this as they had good knowledge of the information regarding the community members in the area. The purpose of the study was explained and the mothers and their significant others in the first 1000 days of life post-conception during the COVID-19 pandemic were recruited from the selected villages. Data was collected between 10^th^ October and 9^th^ November 2020, two months after the first lockdown was eased, during the pandemic in Uganda. Data were collected using individual in-depth, semi-structured interviews. The interviews were scheduled and conducted in the participants’ homes after they had returned from their work (e.g., in gardens/shops). COVID-19 standard operating procedures (SOPs) were strictly observed. Each participant was given a mask and hand sanitizer, and social distancing was always observed. The data collection tool comprised two parts. Part one covered participants’ demographic characteristics including the participant identifier (anonymised name), age, gender, marital status, level of education, number of children, and village. Part two included key open-ended questions regarding participants’ experiences and their understanding of these experiences. All interviews were audio-recorded in Lumasaba, with the transcript later translated into English by a professional translator.

The researcher worked with one other interviewer who could speak the local language. This interviewer completed a 1-day training session, which was critical to ensure the collection of accurate data and adherence to the ethical guidelines.

### Data analysis

The interviews were transcribed verbatim, in the local language (Lumasaba), and then translated to English and entered into the NVivo software package and analysed as they came in until a point of saturation was reached. Analysis was based on the method developed by Braun and Clarke [29]. NVivo was used to generate initial codes for each interview. Thematic analysis was explored, two researchers iteratively read through the interview, developed codes, and grouped them first within each interview and then between the interviews to seek common themes that described the experiences of mothers and their significant others in accessing comprehensive healthcare in the first 1000 days of life post-conception during COVID-19. The entire research team then discussed and amended these initial themes as required. Finally, the main theme and subthemes were agreed upon in another round table discussion following minor amendments.

### Sample characteristics

The mother presented with a relative (significant other) that she considered equally responsible for her healthcare and the healthcare of the children in the family. Eight females; seven mothers participated in the study. One female parent to one of the mothers, and six males; five husbands, and one father-in-law to one of the mothers. The age of the female participants ranged between 23 to 57 years and the males range from 30 to 67 years. Some participants were farmers, and some had small businesses (e.g., shops). Most of the mothers interviewed had no source of independent income yet, and the participants’ family sizes ranged from two to seven children (Table 1).

**Table 1:** Demographic Characteristics of the participants

No Name	Age, years	Gender	Marital Status	Level of education	Employment	Village	No of children	Youngest Child’s age
George	40	Male	Married	Primary 5	Famer	Luyehe B	4	4 months
Alice	23	Female	Married	Primary 7	Housewife	Luyehe B	4	3 months
Joan	32	Female	Married	Primary 1	Farmer	Luyehe B	4	2 months
Sam	35	Male	Married	Primary 5	(Boarder-boarder rider)	Luyehe B	4	3 months
Rose	24	Female	Married	Primary 5	Housewife	Bukasakye	2	18 months
Edward	34	Male	Married	No formal education	Peasant Farmer	. Bukasakye	2	18 months
Mike	30	Male	Married	Primary 7	Businessman	Bukasakye	2	6 months
Justine	25	Female	Married	Primary 3	Housewife	Bukasakye	2	6 months
Sonia	24	Female	Married	Primary 7	Housewife	Makere	3	3 months
Jude	67	Male	Father-in-law	No formal education	Famer	Makere	3	3 months
James	34	Male	Married	Primary 6	Businessman	Makere	7	4 months
Margaret	30	Female	Married	Secondary 4	Housewife	Makere	7	4 months
Norah	57	Female	Widowed Mother to the participant	No formal education	Farmer	Makere	6	5 months
Kaudah	32	Female	Not married	Primary 5	Farmer	Makere	6	5 months

## Results

One theme was identified from the coded and categorized data: The theme captured the ‘Increasing barriers to healthcare. This theme was developed from six subthemes that emerged from an iterative review of the transcripts: accessing healthcare, distressing situations living in fear, making forced choices, navigating the gatekeepers, and ‘Coping with increased poverty. The summary of the results has been presented in Table 2.

**Table 2:** Summary of the results

Quotes	Categories	Subthemes	Themes
It was not easy to move, but I had to go to the local council to look for a letter, the process was not difficult, but again I had to look for transport; meanwhile, the baby’s condition was worsening, the temperature was high, the baby was vomiting, and it had diarrhea. (Edward)	Issues related to transport		
I took care of my daughter, and it was not easy, we had no transport, and they never allowed motorcyclists to carry passengers, they only carried luggage (Norah) I delivered from home because there was no transport, and the motorcyclists refused to take me to the hospital (Kaudah)			
I started labouring during the daytime; unfortunately, we could not access permission to move easily, and transport to the hospital was not readily available. (Margaret)	Missed Health care Opportunities	Access to Health Services	Increasing barriers to healthcare
My wife was supposed to go back to the hospital for review but due to the situation of restricted movement, she did not go until I got permission to move from the Local Council leader. (James)			
My child was sickly and needed constant healthcare. I missed most of the appointments because of the process of getting travel documents and a lack of money. (George)			
I am worried about my baby, I delivered from home, yet I was supposed to deliver from the hospital, he did not get the syrup that prevents HIV in the baby. (Kaudah*)*			
I thought about so many things and then I left everything to God. I knew the baby had to come out. I got prepared to deliver my daughter. Indeed, she pushed the baby to the floor; after delivering, this girl bled almost to death. (Norah)	Access to skilled healthcare providers		
I feel the government could have come up with a solution to this problem, we needed a concrete plan to ensure that care is provided during this difficult time. You see someone should have followed up to see how we were suffering accessing care. (George)			
I remained at home during the time of labour […] It was my mother who said that I can deliver from home… (Kaudah)			
I reached them early in the morning one day at the health facility with my wife, but I left this place late in the evening. The nurses came late, and they left early, I had to wait for the evening nurses. (Sam)			
I have not told you this, but during the COVID-19 pandemic, these children (grandchildren) fell sick [and] I had to go to the drug shop to buy drugs; this to me was very unusual, I have always taken these children to the hospital. The drugs never worked their sickness worsened and I almost lost one. (Jude)	Sickness	Distressing situations	
My wife was supposed to go back to the hospital for review but due to the situation of restricted movement, she did not go until I got permission to move from the Local Council leader. (James)	Missed health care services		
My child was sickly and needed constant healthcare. I missed most of the appointments because of the process of getting travel documents and a lack of money. (George)			
I feel the government could have come up with a solution to this problem, we needed a concrete plan to ensure that care is provided during this difficult time. You see someone should have followed up to see how we were suffering accessing care. (George)			
This time I almost died. There were so many delays…I think they [healthcare workers] are all competent, but none of them came near me, yet I had issues that I wanted to tell the midwife without any other person hearing… (Justine)			
After the very sick child was examined and treated, they told me to go back home which I felt was not right. They gave me 1 month to come back for review. I realised that the health workers ended the clinics prematurely to allow them time to go home early. (George)	Communication between health caregivers and the mothers		
The health workers were not welcoming at all, they kept on telling us to move away, saying that we might infect them with COVID. Indeed, the health workers were harsh on us, and I felt like not going back to the clinic because the love and care I used to receive from the health workers were no longer evident. (Rose)			
The baby remained weak, and we (the husband and his wife) were supposed to go back to the hospital after a week, but I never wanted to go through the same experience again. (Edward)	Previous experiences		
My wife has been seen by the nurses at the health center III I am not sure whether the treatment they offer is appropriate (Mike)	Trust		
But again, another fear that I have at the main hospital they are very strict with people wearing masks. Could it be that there are many people with COVID-19 viruses at this health facility? Here in these facilities around us, it is not a must…It was so scary that at one point had to buy drugs instead of going to the hospital in fear of contracting the coronavirus. (Mike)	Maintaining the Standard Operating Procedures (SOPs)	Living in fear	
These health workers distanced themselves from us. I once had something to tell them, but I could not tell them because I sat very far from the health worker who attended to me, she never even examined me. She asked me questions when everyone was hearing. I just told her that I had a headache, yet I had another issue. They gave me treatment without examining me. (Joan)			
I have a feeling that this daughter-in-law might contract COVID-19 from the health facilities and bring it here because she keeps on telling me that she is going to the health facility […] Can we wait until COVID has subsided then we take the children for immunisation? (Jude)	Stress and uncertainty Fear of contracting COVID 19		
I have a feeling that this daughter-in-law might contract COVID-19 from the health facilities and bring it here because she keeps on telling me that she is going to the health facility […] Can we wait until COVID has subsided then we take the children for immunisation? (Jude)			
I prefer the main hospital Mbale. The only fear I had at that time was that if police found you on the road without a permission letter to move, that would be a crime, and you would risk being beaten up. (Mike)	Alternative sources of service provision and seeking care from lower health facilities	Making forced choices	
My dear friend, this time I had to tell her to go to the health centre near here. I know it is not as good as the other one but due to the challenges in transport and obtaining permission to move she had to go to the nearby health facility. But I think they did a good job; I have not had any complaints from her. (Judi)			
But when I reached the health facility gate, I was chased away because I did not have a mask. The gatekeepers told me to go and wear a mask. (George) VI	Lack of information at the health facility gate	Navigating the gatekeepers	
The other thing I think about is when you go to the hospital for immunisation without a mask, they do not attend to you. They chase you with the baby. The first time I took the baby to the hospital, I had to come back home without the baby being seen. So, I had to go back the following day. (Norah)	Law enforcement without alternative support		
The time I took my child for immunisation, they sympathised with me, and they (the guards) gave me a mask otherwise they were not going to treat my child. (Alice)	Maintaining the restrictive measures		
I did not have money to buy a mask, so I sat at the health facility with my sick child whose condition was worsening each minute that passed until someone gave me a mask and I entered the hospital. (George)	Diminishing resources	Coping with increased poverty	
They requested me to go and look for some drugs that were not available at the clinic, I borrowed the money, and I bought the drug. (Rose)			
I had no money; I had no employment, which is why my sister died. We could not transport her to the hospital to deliver in time. (James)	Financial Implications		
I saw my wife suffer from hunger because I had no money to buy food. We had no money, and the feeding was not the best. (Sam)			
…I did not have money to take care of my sick child, and I could not give my child the food he was meant to eat…This was so hurting, my child’s health deteriorated during this time, and I was lucky that my child did not die. (George)			
…at least I had food I sold all my goats and chicken to support my family during the difficult time of COVID, I thank God I succeeded. (Mike)			

### Theme: increasing barriers to healthcare

#### Subtheme 1: accessing healthcare

Participants’ narratives highlighted how they had experienced difficulty in accessing healthcare. They shared issues related to access to transport to health facilities, missed healthcare opportunities, and the lack of opportunity to receive services from skilled healthcare providers.

##### Issues related to transport

Participants expressed concerns that the restrictions on movement affected their access to healthcare services. They noted that many mothers and their children had missed necessary follow-up care because it was not possible to access transport to healthcare facilities.

*It was not easy to move, but I had to go to the local council to look for a letter, the process was not difficult, but again I had to look for transport; meanwhile, the baby’s condition was worsening, the temperature was high, the baby was vomiting, and it had diarrhea. (Edward)*

*I took care of my daughter, it was not easy, we had no transport, and they never allowed motorcyclists to carry passengers, they only carried luggage (Norah).*

*I delivered from home because there was no transport, and the motorcyclists refused to take me to the hospital (Kaudah)*

The restrictions on travel also meant that some people could not even access local facilities. One participant reported that the worst outcome related to transport issues was that some births were assisted by a layperson or an unskilled service provider, sometimes with poor health outcomes.

* I thought about so many things and then I left everything to God. I knew the baby had to come out. I got prepared to deliver my daughter. Indeed, she pushed the baby to the floor; after delivering, this girl bled almost to death. (Norah)*

A woman who delivered at home with the help of her mother said:

*So I remained at home during the time of labour […] It was my mother who said that I can deliver from home… (Kaudah)*

For one participant, the labour process started at home; the family members had intended to seek help from health facilities, but the process of obtaining permission to travel caused delays.

*I started labouring during the daytime; unfortunately, we could not access permission to move easily, and transport to the hospital was not readily available. (Margaret)*

##### Missed healthcare opportunities

One family identified issues related to missed opportunities to access healthcare. These issues originated from both the family and the health facility. Concerns were expressed about missed healthcare services.

*My child was sickly and needed constant healthcare. I missed most of the appointments because of the process of getting travel documents and a lack of money. (George)*

One participant was delayed in attending antenatal care by the bureaucratic procedures that had been put in place.

*My wife was supposed to go back to the hospital for review but due to the situation of restricted movement, she did not go until I got permission to move from the Local Council leader. (James)*

An HIV-positive mother expressed her continuing fear related to the missed opportunity to save her baby from becoming HIV-positive when breastfeeding because of the lack of follow-up visits.

*I am worried about my baby, I delivered from home, yet I was supposed to deliver from the hospital, he did not get the syrup that prevents HIV in the baby. (Kaudah)*

##### Access to skilled healthcare providers

Among participants who managed to reach a health facility, the shortage of staff and work overload of healthcare workers hindered care provision. In addition, the curfew and lack of transport often meant that the healthcare workers would start their duty late and would leave early despite mothers waiting at the facilities for their services. A related issue was that if a sick child could be taken to a health facility, there were few nurses available to provide care.

*I reached them early in the morning one day at the health facility with my wife, but I left this place late in the evening. The nurses came late, and they left early, I had to wait for the evening nurses. (Sam)*

Participants attributed this situation to the pandemic and recognized the pandemic had contributed to some of the omissions observed at healthcare facilities. But one of the participants lamented that:

*I feel the government could have come up with a solution to this problem, we needed a concrete plan to ensure that care is provided during this difficult time. You see someone should have followed up to see how we were suffering accessing care. (George)*

#### Subtheme 2: distressing situations

Assessing care from qualified personnel became a challenge for participants and caused distress for some. Some participants were forced to buy over-the-counter drugs because of fear of contracting COVID-19 at a health facility or fear of moving around given the restrictions on movement.

*I have not told you this, but during the COVID-19 pandemic, these children (grandchildren) fell sick [and] I had to go to the drug shop to buy drugs; this to me was very unusual, I have always taken these children to the hospital. The drugs never worked their sickness worsened and I almost lost one. (Jude)*

The COVID-19 pandemic affected some health facilities that had to reduce the number of admissions or appointments to cope with the prevailing situation of limited staff. This situation negatively affected participants.

*After the very sick child was examined and treated, they told me to go back home which I felt was not right. They gave me 1 month to come back for review. I realised that the health workers ended the clinics prematurely to allow them time to go home early. (George)*

Many participants expressed dissatisfaction with the services offered at healthcare facilities. They felt that they were neglected because healthcare workers focused on instituting SOPs to prevent cross-infection from COVID 19.

*The health workers were not welcoming at all, they kept on telling us to move away, saying that we might infect them with COVID. Indeed, the health workers were harsh on us, and I felt like not going back to the clinic because the love and care I used to receive from the health workers were no longer evident. (Rose)*

This situation created loneliness among many mothers. One woman explained that before her discharge, she felt alone following her traumatic experience.

*This time I almost died. There were so many delays…I think they [healthcare workers] are all competent, but none of them came near me, yet I had issues that I wanted to tell the midwife without any other person hearing… (Justine)*

Some participants’ experiences at the health facilities negatively affected their intention to access care in the future.

*The baby remained weak, and we (the husband and his wife) were supposed to go back to the hospital after a week, but I never wanted to go through the same experience again. (Edward)*

#### Sub-theme 3: living in fear

Participants and their families described a fear of contracting COVID-19 from health facilities. This fear often resulted in participants missing or delaying necessary healthcare.

*I have a feeling that this daughter-in-law might contract COVID-19 from the health facilities and bring it here because she keeps on telling me that she is going to the health facility […] Can we wait until COVID has subsided then we take the children for immunisation? (Jude)*

In particular, older family members who would normally provide childcare were concerned about the risks of COVID-19 infection themselves:

*I had warned her (daughter-in-law) never to move to the hospital with my grandchildren. I am the one who carries these children when this woman moves away from home, now imagine what if they contract the disease and they pass it on to me at this age, don’t you think I will die? (Jude)*

The rules about mask-wearing in the hospital caused anxiety for some participants, as it emphasised the risk of contracting COVID-19 in the hospital. For these participants, this implied that the hospital should be avoided, and they chose to self-medicate instead.

*But again, another fear that I have at the main hospital they are very strict with people wearing masks. Could it be that there are many people with COVID-19 viruses at this health facility? Here in these facilities around us, it is not a must…It was so scary that at one point had to buy drugs instead of going to the hospital in fear of contracting the coronavirus. (Mike)*

Participants reported that healthcare staff was also noticeably cautious about being exposed to COVID-19. These fears meant that healthcare workers kept their distance from their patients, which reduced the opportunity for clinical examination and confidential discussions.

*These health workers distanced themselves from us. I once had something to tell them, but I could not tell them because I sat very far from the health worker who attended to me, she never even examined me. She asked me questions when everyone was hearing. I just told her that I had a headache, yet I had another issue. They gave me treatment without examining me.* (Joan)

Participants felt that they did not receive appropriate healthcare because of the healthcare providers’ fear of contracting COVID-19 from their patients, which noticeably reduced the level of care provided.

*My wife has been seen by the nurses at the health center III I am not sure whether the treatment they offer is appropriate (Mike)*

#### Subthemes 4: making forced choices

The restrictions on personal and public transport affected participants’ choices about the level of health facility they could attend.

*I prefer the main hospital Mbale. The only fear I had at that time was that if police found you on the road without a permission letter to move, that would be a crime, and you would risk being beaten up. (Mike)*

Other participants were motivated to attend services at a higher-level facility (e.g., HCIV), where they would be offered all treatment free of charge. However, the restrictions implemented because of the pandemic hindered their access to these health facilities, meaning they had to attend a lower-level facility with fewer resources.

*My dear friend, this time I had to tell her to go to the health centre near here. I know it is not as good as the other one but due to the challenges in transport and obtaining permission to move she had to go to the nearby health facility. But I think they did a good job; I have not had any complaints from her. (Judi)*

#### Subtheme 5: navigating the gatekeepers

Participants described how the security guards (literal gatekeepers) at the entrance to the hospital insisted on patients and visitors wearing masks. They reported that little support or advice was offered to those without masks.

*But when I reached the health facility gate I was chased away because I did not have a mask. The gatekeepers told me to go and wear a mask. (George)*

*The other thing I think about is when you go to the hospital for immunisation without a mask, they do not attend to you. They chase you with the baby. The first time I took the baby to the hospital, I had to come back home without the baby being seen. So, I had to go back the following day. (Norah)*

However, they noted that help was sometimes provided by these gatekeepers.

*The time I took my child for immunisation, they sympathised with me, and they (the guards) gave me a mask otherwise they were not going to treat my child. (Alice)*

#### Subtheme 6: coping with increased poverty

Participants shared many issues that arose from the increasing levels of poverty in their communities, which had worsened during the pandemic. The pandemic exacerbated the difficulties caused by poverty that were already being experienced. Some participants had lost their jobs and others could not sell their products to boost their family income. In addition, the added costs of transport and masks had serious impacts on access to healthcare.

*I did not have money to buy a mask, I sat at the health facility with my sick child whose condition was worsening each minute that passed until someone gave me a mask and I entered the hospital. (George)*

*I had no money; I had no employment, which is why my sister died. We could not transport her to the hospital to deliver in time. (James)*

Some participants had borrowed money to buy drugs with no plans for how they could pay the borrowed amount back.

*They requested me to go and look for some drugs that were not available at the clinic, I borrowed the money, and I bought the drug. (Rose)*

Another problem that was reported to be created by poverty and reduced family income was the lack of appropriate nutrition. Some participants described how they had no food to feed their families, a situation that resulted in poor pregnancy and breastfeeding outcomes.

*I saw my wife suffer from hunger because I had no money to buy food. We had no money, and the feeding was not the best. (Sam)*

*…I did not have money to take care of my sick child, and I could not give my child the food he was meant to eat…This was so hurting, my child’s health deteriorated during this time, and I was lucky that my child did not die. (George)*

Other participants shared how they had sold their livestock to buy food as a short-term measure.

*…at least I had food I sold all my goats and chicken to support my family during the difficult time of COVID, I thank God I succeeded. (Mike)*

However, short-term solutions such as selling livestock were likely to have long-term impacts on the family.

## Discussion

This study described the experiences of mothers and their significant others in accessing comprehensive healthcare in the first 1000 days of life post-conception during COVID-19 in rural Uganda. Major barriers to accessing healthcare stood out in participants’ narratives. The lack of access to healthcare was influenced by the restrictive measures implemented by the Government of Uganda to minimise exposure to COVID-19 [30]. These measures presented numerous challenges related to access to health facilities by pregnant mothers and their significant others. There were missed healthcare opportunities, as participants could not attend appointments due to the length of time it took to secure travel documents from local council leaders and other relevant authorities. Previous studies indicate that other Sub-Saharan countries (e.g., Kenya, Nigeria, and South Africa) experienced similar problems in accessing health facilities [, 16, 31–33]. Follow-up care was disrupted in many of these countries, as the emphasis was placed on emergency services [34]. To alleviate this situation in Uganda, emergency services were focused on maternal and child health. Although travel by private car was banned, in the urban community especially Kampala the Capital City of the country, mothers in labour were encouraged to contact their local community leaders for ambulances to take them to a healthcare facility [10]. However, the rural community had limited knowledge regarding the availability of ambulances as the results showed that the mother and their significant others never mention anything concerning the ambulances, yet they were available. This finding highlighted that mothers in rural communities did not access ambulance services. Biryabarema [35] explained that given the scarcity of public ambulances, expectant mothers reached hospitals in hired private vehicles with special permits issued by resident district commissioners, although these authorities were frequently inaccessible to community members. In addition, the existing poverty exacerbated by the pandemic meant that many families could not afford to hire transport. Inevitably, the bureaucratic procedures required to gain permission (e.g., letters) to travel to health facilities delayed mothers from accessing these facilities [36].

There is some evidence to suggest that participants in this study had mixed feelings regarding the quality of care they received from healthcare providers. Some participants described the actual and apparent shortage of health workers because, they did not want to draw near to the mothers and their families for fear of contracting the virus, and others did not give attention to participants’ concerns. Consequently, mothers spent long hours at the health facilities. Such experiences are likely to create a continuing barrier to accessing comprehensive healthcare [31, 36]. Other studies indicated these shortcomings in the provision of care were caused by challenges in traveling to and from workplaces and no assistance provided to reach health facilities [33, 37].

Fear of infection was evident among participants, which was related to their knowledge about their safety at health facilities. Other studies reported similar findings regarding fear among mothers and their families of acquiring and passing on COVID-19 infection to family members [38]. This fear may also have contributed to delays in seeking healthcare.

Participants in that study preferred delivering at the main hospital because of fear of contracting COVID-19 from lower-level health facilities. The findings of this study showed that participants’ preferences about attending maternal and child healthcare were altered by the pandemic, with mothers having no other option but to seek care from the lower-level facilities available nearby yet, they expressed dissatisfaction with the care received from these health facilities. Similar findings were reported by [39], who explored mothers’ experiences of accessing maternal health services with the preference of delivering from higher-level facilities.

Gatekeepers at the health facilities ensured that all visitors wore a mask before entering the health faculties. This caused anxiety among mothers and their families and meant that some avoided visiting health facilities. Some participants shared that their financial status meant they could not afford to buy a mask, so they returned home without accessing care. This situation was also reported in a study from Bangladesh by Koustuv who aimed to understand the perception and practices of COVID-19 infection prevention. That study showed that because of poverty, community members could not afford to buy masks despite understanding the importance of masks. In this study, participants reported existing poverty was exaggerated by COVID-19, which affected their decisions to access healthcare. This situation was also reported by Roberton 2020 who discussed coverage of essential maternal and child health interventions in low and middle-income countries. Those authors reported that maternal and newborn mortality had increased because of the lack of access to healthcare services created by poverty and the COVID-19 pandemic.

The present findings indicated that there were consequences of restricted access to health facilities The delays in accessing healthcare and the omissions described indicate that there were missed healthcare opportunities, as some mothers missed delivering with the help of skilled healthcare workers, whereas others were discouraged by unpleasant experiences at the health facilities where they had to wait for long hours because of staff shortages. Similarly, the number of times mothers visited health facilities for antenatal services, was reduced and some mothers had to deliver at home with sometimes traumatic outcomes. As our findings showed, one participant shared that she had almost bled to death after she had given birth because she could not access professional healthcare services in time. In addition, children with severe health conditions reached health facilities late and others missed out on important healthcare, such as immunisation and access to antiretroviral drugs This was supported by Amimo, Lambert, and Magit [40] who reported difficulties in accessing antiretroviral and prophylactic drugs among people living with HIV and AIDS. Similar situations were evident from other research, even in developed countries where care was perceived as impersonal and inadequate despite having resources available [41–43]. However, in developing countries such as Uganda, maternal and child health was grossly affected by increased mortality among newborns early in the COVID-19 pandemic [44, 45]. This highlighted that COVID-19 exacerbated healthcare services and supplies. A comparable experience was reported in Bangladesh where institutional childbirth was reduced and stillbirths increased because of COVID-19 restrictions [45].

This situation contradicts current maternal and child health guidelines that advocate for mothers to attend antenatal visits and mothers to give birth at health facilities with the help of trained healthcare providers [46]. A previous study also noted that old questions, such as where to give birth, had resurfaced in the context of COVID-19 [16]. Ugandan HCIII facilities are geared towards providing care for those expected to have normal births, which aims to prevent hospitals from becoming overfull; however, participants’ narratives suggested that accessing care at the facilities was difficult. This was also reflected in previous studies [47, 48], which explained how maternity health services were experiencing added stress due to COVID-19 and highlighted that patients and healthcare workers required more support [49] also reported that there was a need for extra psychological support during the pandemic as pregnant women were reporting increased levels of depression and anxiety. This was partly attributed to women facing uncertainty about adequate access to healthcare services.

## Conclusion

This study shows that the global pandemic increased barriers to healthcare services in rural Uganda. This rural population experienced difficulties in accessing health services, along with the lack of access to expert care given the shortage of skilled healthcare workers. In some cases, this led to mothers having to give birth without skilled help, which may increase maternal and child morbidity and mortality. Bureaucratic procedures put in place because of COVID-19 also led to travel barriers, and the lack of aid and medical resources increased fears among community members. Furthermore, some who were treated at health facilities had traumatic experiences, which may prevent them from returning for further treatments, including immunisation. Moreover, the lack of effective communication between different levels of health services may prevent such fears from being heard and addressed.

### Recommendations

There is a need to identify ways of improving access to healthcare and communication during pandemics or similar emergencies in Uganda using available means. COVID-19 amplified pressure on the already strained healthcare services in Uganda with the referral chain only able to move up one level at a time. This calls for the government to support and empower midwives and bring these services closer to communities. It is also necessary to strengthen community- and home-based care.

If another lockdown is implemented in the future, HCs at lower levels need to be available near the communities where mothers live so that those without anticipated complications can deliver skilled help. Secondly, the gap between the supply of equipment and consumables like Personal Protective Equipment and their demand during a pandemic should be predicted and bridged in time to promote healthcare services at the lower level of facilities. Similarly, lessons learned from the current COVID-19 study could be used in the Ebola epidemic current in Uganda. Finally, further projects may consider investigating the potential of upskilling some members of the healthcare team in lower-level facilities, such as village healthcare teams and traditional birth attendants.

## Guidelines followed

Potential participants were accessed through local leaders in Mbale Mutoto after they received an explanation of the study’s objective. The local leaders with the help of the HVTs then guided the researchers to potential participants, who had all the required information regarding the citizens in the selected study area. Pregnant mothers or those with infants up to two years (first 1000 days of life) and significant others in the family from Bunghokho-Mutoto Sub-County, Mbale, were recruited for this study after consenting. Participants were purposively selected from villages that were more than 10 km from the main district hospital as these community residents may have faced challenges assessing the main facility. These villages included Luyehe B, Bukasakye, Bunamwani, and Makere. All data are fully available without restriction.

## Supplementary Information

**Additional file 1.**

**Additional file 2.**

**Additional file 3.**

**Additional file 4.**

**Additional file 5.**

**Additional file 6.**

**Additional file 7.**

**Additional file 8.**

**Additional file 9.**

**Additional file 10.**

**Additional file 11.**

**Additional file 12.**

**Additional file 13.**

## Data Availability

The interview transcriptions have been uploaded as supplementary files.

## References

[1] World Health Organization. Coronavirus disease 2019 (COVID-19): Situation Report, 73. 2020. https://apps.who.int/iris/bitstream/handle/10665/331686/nCoVsitrep02Apr2020-eng.pdf. Available from. [Cited 20 Oct 2022]

[2] Ario AR, Muruta AN, Mbaka P, Bulage L, Kwesiga B, Kadobera D, Morukileng J, Emong JD, Akusekera I. MINISTRY OF HEALTH UGANDA. Epidemiol Bull. 2020;5(3). https://uniph.go.ug/wp-content/uploads/2021/07/UNIPH-Bulletin_Issue-3-Volume-5.pdf.

[3] Mukunya D, Tumwine JK (2020). Editorial ii Challenges of tackling non COVID-19 emergencies during the unprecedented pandemic. Afr Health Sci.

[4] Mizumoto K, Kagaya K, Zarebski A, Chowell G (2020). Estimating the asymptomatic proportion of coronavirus disease 2019 (COVID-19) cases on board the Diamond Princess cruise ship, Yokohama, Japan, 2020. Euro Surveill.

[5] Kiwanuka F, Waswa S, Alemayehu YH, Simbeye JA (2020). Policy decisions and response to fight 2019 novel coronavirus disease in Uganda: a review of attributes, comprehensiveness, and implications to improve resilience to future pandemics.

[6] Ministry of Health. Situation analysis of newborn health in Uganda: Current status and opportunities to improve care and survival. https://www.healthynewbornnetwork.org. Available from. Cited 20 Jun 2022

[7] Burt JF, Ouma J, Lubyayi L, Amone A, Aol L, Sekikubo M, Nakimuli A, Nakabembe E, Mboizi R, Musoke P, Kyohere M (2021). Indirect effects of COVID-19 on maternal, neonatal, child, sexual and reproductive health services in Kampala, Uganda. BMJ Glob Health.

[8] Katana E, Amodan BO, Bulage L, Ario AR, Fodjo JNS, Colebunders R, Wanyenze RK (2021). Violence and discrimination among Ugandan residents during the COVID-19 lockdown. BMC Public Health..

[9] Beluska-Turkan K, Korczak R, Hartell B, Moskal K, Maukonen J, Alexander DE, Salem N, Harkness L, Ayad W, Szaro J, Zhang K (2019). Nutritional gaps and supplementation in the first 1000 days. Nutrients..

[10] Mizuno Y, Kagitani-Shimono K, Jung M, Makita K, Takiguchi S, Fujisawa TX, Tachibana M, Nakanishi M, Mohri I, Taniike M, Tomoda A (2019). Structural brain abnormalities in children and adolescents with comorbid autism spectrum disorder and attention-deficit/hyperactivity disorder. Translational psychiatry..

[11] World Health Organisation (2005). Annual report: making every mother and child count.

[12] Kahn JA, Larson G. Pathfinder Minneapolis, MN. Educational Considerations. 17(1);9:31. 10.4148/0146-9282.1570.

[13] Rutaremwa G, Wandera SO, Jhamba T, Akiror E, Kiconco A (2015). Determinants of maternal health services utilization in Uganda. BMC Health Serv Res.

[14] Kawungezi PC, AkiiBua D, Aleni C, Chitayi M, Niwaha A, Kazibwe A, Sunya E, Mumbere EW, Mutesi C, Tukei C, Kasangaki A (2015). Attendance and utilization of antenatal care (ANC) services: multi-center study in upcountry areas of Uganda. Open J Prev Med.

[15] Atuhaire S, Mugisha JF (2020). Determinants of antenatal care visits and their impact on the choice of birthplace among mothers in Uganda: a systematic review. Obstet Gynecol Int J.

[16] Bukuluki P, Kisaakye P, Mulekya F, Mushomi J, Mayora C, Palattiyil G, Sidhva D, Nair H (2022). Disruption in accessing sexual and reproductive health services among border populations during COVID-19 lockdown in Uganda. J Glob Health..

[17] Brazzell JF, Acock AC (1988). Influence of attitudes, significant others, and aspirations on how adolescents intend to resolve a premarital pregnancy. J Marriage Fam.

[18] Sell M, Minot N (2018). What factors explain women's empowerment? decision-making among small-scale farmers in Uganda. Womens Stud Int Forum.

[19] Ministry of Health. Situation analysis of newborn health in Uganda: Current status and opportunities to improve care and survival. https://www.healthynewbornnetwork.org. Retrieved 20 Jun 2022

[20] Chattu VK, Yaya S (2020). Emerging infectious diseases, and outbreaks: implications for women’s reproductive health and rights in resource-poor settings. Reprod Health.

[21] Esegbona-Adeigbe S. Impact of COVID-19 on antenatal care provision. European Journal of Midwifery. 2020;4. 10.18332/ejm/121096 PMC7839116 33537618

[22] Hug L, Mishra A, Lee S, You D, Moran A, Strong KL, Cao B. A neglected tragedy The global burden of stillbirths: Report of the UN inter-agency group for child mortality estimation. London School of Hygiene & Tropical Medicine; and Allisyn Moran at the World Health Organization (WHO). 2020. [cited 2022 Nov 3]. Available from: http://202.1.196.72/jspui/bitstream/123456789/8668/1/UN-IGME-the-global-burden-of-stillbirths-2020.pdf

[23] Pallangyo E, Nakate MG, Maina R, Fleming V (2020). The impact of covid-19 on midwives’ practice in Kenya, Uganda, and Tanzania: a reflective account. Midwifery.

[24] Colorafi KJ, Evans B (2016). Qualitative descriptive methods in health science research qualitative descriptive methods in health science research. HERD.

[25] The Republic of Uganda, (2015). Hazard, Risk, and Vulnerability Profile Mbale District. https://docplayer.net/196597465-Mbale-district-hazard-risk-and-vulnerability-profile.html. Retrieved on 24 Oct 2022

[26] Jakubowski A, Egger D, Mulebeke R, Akankwasa P, Muruta A, Kiwanuka N, Wanyenze R. Evaluation of a national program to distribute free masks for COVID-19 prevention In Uganda: Evidence from Mbale District. CEGA Working Paper Series No. WPS-193. Center for Effective Global Action. University of California, Berkeley. Text. 10.5072/FK26Q22802.

[27] Mbulamani J (2018). Mothers Heart Uganda Mutoto Mbale.

[28] Boddy CR. Sample size for qualitative research. Qual Market Res Int J. 2016;4;426–32. 10.1108/QMR-06-2016-0053.

[29] Clarke V (2006). Using thematic analysis in psychology. Qual Res Psychol.

[30] Armbruster S, Klotzbücher V. Lost in lockdown? COVID-19, social distancing, and mental health in Germany. Diskussionsbeiträge; 2020;4. https://www.econstor.eu/bitstream/10419/218885/1/1698957106.pdf

[31] Kassie A, Wale A, Yismaw W (2021). Impact of coronavirus Diseases-2019 (COVID-19) on utilization and outcome of reproductive, maternal, and newborn health services at governmental health facilities in South West Ethiopia, 2020: a comparative cross-sectional study. Int J Women's Health.

[32] Kimani R, Maina R, Shumba C, Shaibu S (2020). Maternal and newborn care during the COVID-19 pandemic in Kenya: re-contextualizing the community midwifery model. Hum Resour Health.

[33] Ombere SO. Access to maternal health services during the COVID-19 pandemic: Experiences of indigent mothers and health care providers in Kilifi County, Kenya. Front Soc. 2021;6:613042. 10.3389/fsoc.2021.613042. 10.3389/fsoc.2021.613042 PMC8058360 33898553

[34] Joska J, Andersen L, Rabie S, Marais A, Ndwandwa E, Wilson P (2020). COVID-19: Increased risk to the mental health and safety of women living with HIV in South Africa. AIDS and Behaviour.

[35] Biryabarema, E. (2020). In Uganda, mothers in labour die amidst coronavirus lockdowns. US News.

[36] Aranda Z, Binde T, Tashman K, Tadikonda A, Mawindo B, Maweu D, Boley EJ, Mphande I, Dumbuya I, Montaño M, Clisbee M (2022). Disruptions in maternal health service use during the COVID-19 pandemic in 2020: Experiences from 37 health facilities in low-income and middle-income countries. BMJ Global Health..

[37] Koustuv D, Saidur RM, Abu SA, Animesh B, Tasnuva H (2021). Community understanding, perception and practices on infection prevention from the coronavirus disease (covid-19): a qualitative study in rural BANGLADESH. Interdiscip Approach Med.

[38] Roberton T, Carter ED, Chou VB, Stegmuller AR, Jackson BD, Tam Y, Walker N (2020). Early estimates of the indirect effects of the COVID-19 pandemic on maternal and child mortality in low-income and middle-income countries: a modeling study. Lancet Global Health..

[39] Vasilevski V, Sweet L, Bradfield Z, Wilson AN, Hauck Y, Kuliukas L, Homer CS, Szabo RA, Wynter K (2022). Receiving maternity care during the COVID-19 pandemic: Experiences of women’s partners and support persons. Women and Birth.

[40] Amimo F, Lambert B, Magit A (2020). What does the COVID-19 pandemic mean for HIV, tuberculosis, and malaria control?. Trop Med Health.

[41] Kotlar B, Gerson E, Petrillo S, Langer A, Tiemeier H (2021). The impact of the COVID-19 pandemic on maternal and perinatal health: a scoping review. Reprod Health.

[42] Hedstrom A, Mubiri P, Nyonyintono J, Nakakande J, Magnusson B, Vaughan M, Waiswa P, Batra M (2021). Impact of the early COVID-19 pandemic on outcomes in a rural Ugandan neonatal unit: A retrospective cohort study. PloS one..

[43] Hogan AB, Jewell BL, Sherrard-Smith E, Vesga JF, Watson OJ, Whittaker C, Hamlet A, Smith JA, Winskill P, Verity R, Baguelin M. Potential impact of the COVID-19 pandemic on HIV, tuberculosis, and malaria in low-income and middle-income countries: a modelling study. The Lancet Global Health. 2020;8(9):e1132-41. 10.1016/S2214-109X(20)30288-6. 10.1016/S2214-109X(20)30288-6 PMC7357988 32673577

[44] Rahman M, Halder H, Islam M (2021). Effects of COVID-19 on maternal institutional delivery: fear of a rise in maternal mortality. J Glob Health.

[45] World Health Organisation (2016). Standards for improving the quality of maternal and newborn care in health facilities.

[46] Semaan A, Audet C, Huysmans E, Afolabi B, Assarag B, Banke-Thomas A, Blencowe H, Caluwaerts S, Campbell OM, Cavallaro FL, Chavane L (2020). Voices from the frontline: findings from a thematic analysis of a rapid online global survey of maternal and newborn health professionals facing the COVID-19 pandemic. BMJ global health..

[47] I’mAtim MG, Kajogoo VD, Amare D, Said B, Geleta M, Muchie Y, Tesfahunei HA, Assefa DG, Manyazewal T (2021). COVID-19 and health sector development plans in africa: the impact on maternal and child health outcomes in Uganda. Risk Manag. Healthc. Policy.

[48] Durankuş F, Aksu E (2022). Effects of the COVID-19 pandemic on anxiety and depressive symptoms in pregnant women: a preliminary study. J Matern Fetal Neonatal Med.

[49] Wu Y, Zhang C, Liu H, Duan C, Li C, Fan J, Li H, Chen L, Xu H, Li X, Guo Y (2020). Perinatal depressive and anxiety symptoms of pregnant women during the coronavirus disease 2019 outbreak in China. Am J Obstet Gynecol.

