# Supplementary material for: Experiences of mothers and significant others in accessing comprehensive healthcare in the first 1000 days of life post-conception during COVID-19 in rural Uganda
Source: BMC Pregnancy Childbirth. 2022 Dec 15;22:938. doi: 10.1186/s12884-022-05212-x (PMC9754309; doi:10.1186/s12884-022-05212-x)
Supplement: Supplementary file 1 — Additional file 1. [file 12884_2022_5212_MOESM1_ESM.docx]

**Interview Guide for the Women and their significant others**

**Anonymised Identifier: Alice**

**Title of the Study:**

Experiences of social isolation and social distancing for women and the significant others in the family on continuity of care in the first 1000 days of life during the COVID 19 pandemic at Bunghokho-Motto Sub-county Mbale.

**Personal information**

**Identification: Alice**

Tell me more about yourself

**Gender**: Female

**Age:** 23years

**Address:** Luyehe B

**Marital status**: Married

**No. Children**: 4

**Young child**: 3 months

**Educational level**: P7

**Interviewer G:** What has been your experience of being cared for/care provided to the pregnant woman or infant in your family during the time of the pandemic?

**Alice:** During the time of the covid pandemic, I experienced a very difficult time. My husband is a Boda Boda (motorist) rider and this time he was not working life became so difficult (cried). We had to get help from the neighbors because my children were staving. We moved to settle here from the landslide area, …. We have just settled here our food had not yet grown to sustain us. It is hard to depend on the mercy of other people.

When I went to visit the health facility for antenatal care the first time, they told me that my baby was not growing well. They said that it was very small

**Interviewer G**: What did you understand by the issue of the baby being very small?

**Alice:** You know they told me that the baby was small and yet I was about to deliver.

**Interviewer G:** How many months was the pregnancy then?

**Alice:**  It was about 8 months because from that time I delivered after one and a half months… I think it was 8 months.

**Interviewer G**: At delivery, had the baby grown up to full-term?

**Alice:** I think it had grown, it was very small at the time of birth but it somehow cried

**Grace:** What was the weight of the baby?

**Alice**: The baby weighed 2 kgs.

**Interviewer G:** How many kg did the previous baby weigh

**Alice:** I remember it was 3.5 kgs, but this baby was small even at a glance “*it took*”, it was a bit weak, it cried but not as the previous babies.

**Interviewer G: Did they tell you what the baby scored,?**

**Alice:** No, nobody talked about that eee what is that?

**Grace:** OK let me have a look at the discharge form for this baby.

**Alice:** Participant: It is here.

**Interviewer G:** Ok, this is the score to show how well your baby is at birth. Your baby scored 7 thank you for sharing. The score is less that what is required; score 10 is the best that means the had some issues that needed immediate care

**Alice**: Aaa no but the baby was small. And the midwife said that I was aaaa.. because I did not feed well during pregnancy.

**Interviewer G**: What happened after you delivered?

**Alice**: We were discharged. My husband brought me back home in the evening because I was delivered in the morning. All the midwives we going away. you know this was covid time. When we reached home still we had very little food, I thank my neighbors who supported me all that time. My major problem at that time was the baby crying all the time. Whenever I put the baby on the breast it would just cry,; .. there was no breast milk. You just look at this baby is still small compared to the siblings.

**Interviewer G**: Sorry about that. Do you still have a problem with breast milk?

**Alice** : Aaa not really, but I feel my baby is not getting enough milk, I am about to start giving it porridge.

**Interviewer G:** Have you gone to see a medical person to raise this problem**.**

No, I do not think they can do anything to help me. The problem is the low income, it has not yet stabilized, my husband is straggling with the family and yet I am not working.

**Interviewer G: If** COVID-19 had not happened where would you be seeking health care?

If covid had not been there I would have gone to the major hospital, … Mbale hospital.

**Interviewer G**: Why?

**Alice:** At the major hospital, they do not leave you to go immediately after you deliver. Remember I was weak, but I had to travel 3 km back home. With my baby. My husband helped me.

**Interviewer G:** How has this changed from before**?**

**Alice**: Things have changed at the health facilities, if you do not have a mask nobody touches you, nobody allows you in the health facility without a mask. Before we used to go to the hospital without masks. I think this was the same issue even at the main hospital. The time I took my child for immunization, they sympathized with me and they gave me a mask otherwise they were not going to treat my child. ….I always delivered from the main hospital, this time I could not reach there on foot, it is very far from here.

**Interview G:** How far is it?

**Alice**: It is 15 miles from here, and there was no transport at that time. There were so many issues that I don’t want to talk about now.

**Interviewer G:** How many times have you taken your child for immunization?

**Alice:** I have gone for immunization two times. After I delivered the midwife told me then I should wait for the person who would immunize my child, but by the time we left this nurse had not come yet. I had to go back after two weeks, remember I was weak. That time my child was immunized. I also went back the second time. But now I have not yet gone back.

**Interviewer G:** Who decides on the care the baby and yourself get in this family?

**Alice:** It is my husband because I do not work he is the one to tell me that now he has money we can go to the hospital. Yes, he is a good man.

**Interviewer G:** What impact do you feel these changes have had on your care/the care?

During pregnancy, I was always falling sick, j used to take herbs, I went for antenatal for only three times because I knew the midwives at the Health Centre III, this is the facility near hear would refer me to the main hospital, I knew I would not go there. So I treated myself and I waited for the time that I would go to deliver my baby

**Interviewer G:** Do you feel confident with the care provision you/the woman or infant received?

**Alice:** I delivered from Health Center III. These midwives were so good they took care of me and handled me well as compared to the main hospital, only that the room where I delivered was too small, we were two mothers delivering at the same time. This time they had very few mothers who had come to deliver. Let me tell you there is a Traditional Birth attendant near my home, she used to invite me to her place but I refused, I feared going to her place because the midwives had already told me the baby was very small.

**Interviewer G:** Do you think any other measures could have been taken to help you?

**Alice:** No

**Interviewer G:** Did you or do you receive advice/care from any informal carers? If so, who?

**Alice**: Nobody talked to me other than the midwives

**Interivewer G:** What fears/ concerns do you now have?

**Alice**: But if this situation continues my husband will not be able to take care of us due to poverty and our health workers will stop helping us because they fear contracting the disease from us.

**Interviewer G**: How do you feel about your baby’s health?

**Alice:** My dear, I am not happy; my baby is small, I think it is because I have little breast milk and when I raised this concern In the hospital they told me to eat these small fish (silverfish) and greens, I have tried with limited success.

**Interivewer G**: Thank you for interacting with us I will talk to you about breastfeeding.
