# Supplementary material for: Experiences of mothers and significant others in accessing comprehensive healthcare in the first 1000 days of life post-conception during COVID-19 in rural Uganda
Source: BMC Pregnancy Childbirth. 2022 Dec 15;22:938. doi: 10.1186/s12884-022-05212-x (PMC9754309; doi:10.1186/s12884-022-05212-x)
Supplement: Supplementary file 2 — Additional file 2. [file 12884_2022_5212_MOESM2_ESM.docx]

## .    **Interview Guide for the Women and their significant others**

**Anonymised Identifier: Edward**

**Title of the Study:** Experiences of social isolation and social distancing for women and the significant others in the family on continuity of care in the first 1000 days of life during the COVID 19 pandemic at Bunghokho-Motto Sub- County Mbale.

**Personal information**

**Identification: Edward**

Tell me more about yourself.

1. **Work**: Peasant
2. **Age**: 34
3. **Gender**: Male
4. **Address**: Bukasakye
5. **Marital status**: Married
6. **Family**: 2 children
7. **Youngest child**: 18 months
8. Education background: No formal Education

**Interviewer G:** What has been your experience of being cared for/care to a pregnant woman, labouring, postnatal, or infant during the time of the pandemic?

**Edward**: I have not been working for long, during the pandemic it worsened. Our child once fell sick in April and we had to take her to the health facility. It was not easy to move. But I had to go to the LC to look for a letter, the process was not difficult, but again I had to look for transport, meanwhile, the baby’s condition was worsening, the temperature was high, the baby was vomiting, and it had diarrhoea. I managed to reach the hospital but again I did not move with a mask, I decided to tie my handkerchief on my mouth, I only bought one mask for my wife. The baby was taken care of well, but they never told what the baby was suffering from, I feared asking them as they were busy all the time.

**Interviewer G**: What was done to the baby?

Edward Ooo, I saw the doctor and the nurses moving up and down, I don’t know what they were doing. They put up a drip, and some drugs were injected in the bottle hanged up. They requested me to go and look for some drugs that were not available at the clinic, borrowed money, and bought it. The child recovered well and we were discharged home after one week. The baby remained a week, we were supposed to go back to the hospital after one week but I never wanted to go through the same experience again.

**Interviewer G:** If COVID-19 had not happened where would you be seeking health care?

**Edward:** I used to take my wife and the baby to Health Center IV (HCIV), the treatment is the same as what is offered at HCIII. But of recent, I have realized that the nurses at this HCIII are slow as compared to the HCIV. Yet HCIV has more patients but the nurses move faster.

**Interviewer G:** How has this changed from before?

**Edward:** I cannot go to the HCIV all the time, it is far, but that time when my child was sick, I had to take the child to this health center. Now with this woman’s pregnancy, we have been going to the HCIII. She will tell you how they have taken care of her.

**Interviewer G:** Who has initiated the changes?

**Edward:** We make decisions together with my wife, I cannot decide for her, ……she decides where she wants to go for treatment or treat her child. What she decides to do what we do. The only problem I have is the limited income.

**Interviewer G:** What impact do you feel these changes have had on your care/ on the care to a pregnant woman, labouring, postnatal, or infant?

**Edward:** My family has not been affected by the Covid pandemic; it is only the process of getting permission to move was the major issue. I just imagine, look at her if she is laboring now and I have to go and seek permission to move, will the baby wait for me to come back with the letter and transport before it comes? This is not so clear to me. Maybe the other issue is that the baby did not get the last immunization, the mother was told last week. I am waiting for her to deliver this one and I take them at the same time.

**Interviewer G:** Do you feel confident about the care provider you received?

**Edward:** I trust them, but they are a bit slow. I have already told you that the ones at Health Centre IV move faster.

**Interviewer G:** Thank you for participating in the study

.
