# Supplementary material for: Experiences of mothers and significant others in accessing comprehensive healthcare in the first 1000 days of life post-conception during COVID-19 in rural Uganda
Source: BMC Pregnancy Childbirth. 2022 Dec 15;22:938. doi: 10.1186/s12884-022-05212-x (PMC9754309; doi:10.1186/s12884-022-05212-x)
Supplement: Supplementary file 3 — Additional file 3. [file 12884_2022_5212_MOESM3_ESM.docx]

##

## Interview Guide for the Women and their significant others

**Anonymised Identifier:** George:

**Title of the Study:**

Experiences of social isolation and social distancing for women and the significant others in the family on continuity of care in the first 1000 days of life during the COVID 19 pandemic at Bunghokho-Motto Sub-county Mbale.

**Interviewer Jalia**

**Personal information**: George

Tell me more about yourself.

1. **Work**: Farmer
2. **Age:** 40 year
3. **Marital status**: Married
4. **Address**: Luyehe Village
5. **Family:** 4 members
6. **Number of children**: 7
7. **Education background**: Primary 5

**Interviewer Jalia:** What has been your experience of the care given to infant during the time of the pandemic?

**George:** During the covid 19 times I had several problems; first I had no money to take care of my family. My child (1 6/12) fell sick during this time and I had to go to the health facility (nearby Hospital) but when I reached the health facility gate I was chested away because I did not have a mask. The gatekeepers told me to go and wear a mask, he added that if your child dies, let it die but you will not enter the hospital without a mask. I did not have money to buy one, I sat at the health facility with my sick child whose condition was worsening each minute that passed until someone gave me a mask and I entered the hospital. All this time my child was in pain because he was a known stickler and his condition was worsening. I was not the only one who was sent away, even other patients were sent away without treatment because they did not have masks. After the child was examined and treated, they told me to go back home which I felt was not right. They gave one month to come back for review. I realized that the health workers ended the clinics early to allow them time to go home early.

**Interviewer Jalia**: If COVID-19 had not happened where would you seek health care for your child?

I would have gone to the Main Mbale Hospital. During this time, I had to go to the Local Council leader for a letter to allow me to move with my child on a motorcycle

**Interviewer Jalia**: How has this changed from before?

**George:** Before covid, I did not have to wear a mask and I was free to move to the hospital without any permission letters from the LC leaders. Then one would take the letter to the sub-county, the here they gave me a letter to the Resident District Commissioner (RDC), this process was so long Second I did not have money to take care of my sick child, I could not give my child the food he was meant to eat … This was so hurting, my child’s health deteriorated during this time, I was lucky that my child did not die. The health workers were not welcoming at all, they kept on telling us to move away, saying that we might infect them with covid. Indeed the health workers were hash on us and I felt like not going back to the clinic because the love and care I used to receive from the health workers were no longer evident.

Remember my child was diagnosed with sickle cell disease, I could meet the appointment dates, worse still these days were spaced. I used to take the child every two weeks for review whenever he fell sick, this time I was given one month. All the same, I missed most of the appointments because of the process of getting travel documents and lack of money.

**Interviewer Jalia**

Who has initiated the changes?

**George:** I can not tell.

**Interviewer Jalia:** Who made the decision to go to the nearby health facility

**George:** I am the man in this family, I make all the decisions

**Interviewer Jalia:** What impact do you feel these changes have had on your child?

**George:** My child’s health has changed, I see as if the child is not growing normally as compared to the other children I have. Second, at times feel like not going back to the hospital. …..What can I do I have to go back to save my child.

**Interviewer Jalia:** What fears/ concerns do you now have?

My fear is that the situation is likely to worsen if the covid pandemic does not end, …. I may lose my child if the health workers continue being rude. Before Covid 19 I was confident with the care that I received from the health workers but now I feel something is missing….. the government should help us.
