# Supplementary material for: Experiences of mothers and significant others in accessing comprehensive healthcare in the first 1000 days of life post-conception during COVID-19 in rural Uganda
Source: BMC Pregnancy Childbirth. 2022 Dec 15;22:938. doi: 10.1186/s12884-022-05212-x (PMC9754309; doi:10.1186/s12884-022-05212-x)
Supplement: Supplementary file 4 — Additional file 4. [file 12884_2022_5212_MOESM4_ESM.docx]

**Interview Guide for the Women and their significant others**

**Anonymised Identifier: James**

**Title of the Study:**

Experiences of social isolation and social distancing for women and the significant others in the family on continuity of care in the first 1000 days of life during the COVID 19 pandemic at Bunghokho-Motto Sub- County Mbale.

**Interviewer G**: Tell me more about yourself.

1. Work, Businessman
2. Age: 34
3. Marital status: Married
4. **Address:** Makere
5. Family: 7 children
6. Youngest Child: 8 months
7. Pregnant women 4 months
8. education background: P6

**Interviewer G**: What has been your experience of being cared for/care to a pregnant woman, labouring, postnatal, or infant during the time of the pandemic?

**James:** I work in Kampala, and I leave this woman here alone with the kid. The good thing was that during the time of covid pandemic there were health workers at the facility who would come to our homes whenever they were called upon therefore, during this time they would come see my wife as she was nearing term they told me to visit the nearby hospital anytime she gets a problem. They examined her here at home and prescribed treatment. It was not that all the time I called them that they came, at times they failed to turn up. Then I would take her on a motorcycle to the health facility.

**Interviewer G**: How far is the health facility from hear?

**James:** It is not far; it is about 1 mile from here

**Interviewer G:** If COVID-19 had not happened where would your pregnant woman, and your family be seeking health care?

**James**: At this same health facility, it is a private health center, it provides all the care I need I do not see why I should move to another health facility.

**Interviewer G:** How has this changed from before?

**James**: I have not changed. This is a private hospital I used to go to the government health facility but they had limited drugs. The other thing was that if you go to the health facility in the morning, you come back in the evening. I could not afford to stay at the health facility for this long. We had no other person to leave home with the baby, it is better I go and pay instead of spending the whole day at the health facility. Though sometimes they would work on us early because I always escorted my wife, but these facilities have no drugs.

**Interview G**: Who makes decision related to health here in your family

**James**: Okay, at times we sit and discuss, but this time it was me making all the decisions because she had no other choice.

**Interviewer G**: What impact do you feel these changes have had on your care/ on the care to a pregnant woman, labouring, postnatal, or infant?

**James**: Apart from me stopping my business at Kampala, covid has not affected me so much because I had some little money that I had kept with me. It has really helped me. But now it is almost done, I need to go back and work.

**Interviewer G:** What fears/ concerns do you now have?

**James**: If this situation continues, the health facilities have no drugs where shall we get treatment especially to these young ones?

**Interviewer G**: Thank you for participating in this study.
