# Supplementary material for: Experiences of mothers and significant others in accessing comprehensive healthcare in the first 1000 days of life post-conception during COVID-19 in rural Uganda
Source: BMC Pregnancy Childbirth. 2022 Dec 15;22:938. doi: 10.1186/s12884-022-05212-x (PMC9754309; doi:10.1186/s12884-022-05212-x)
Supplement: Supplementary file 5 — Additional file 5. [file 12884_2022_5212_MOESM5_ESM.docx]

.    **Interview Guide for the Women and their significant others**

**Title of the Study:** Experiences of social isolation and social distancing for women and the significant others in the family on continuity of care in the first 1000 days of life during the COVID 19 pandemic at Bunghokho-Motto Sub- County Mbale.

Personal**information**

**Identification:** Joan

**Tell me more about yourself**.

1. Work: House wefe
2. Age: 32 yrs
3. Marital status: Married
4. **Address**: Luyehe B
5. Family Members: 4
6. Youngest child 2 years
7. Education background: P1

**Interviewer Jalia:**

1. What has been your experience of being cared for/care to a pregnant woman, laboring, postnatal, or infant during the time of the pandemic?

**Joan:** During the covid time I moved with my child who was diagnosed with sickle cell disease to many places. I move to the Health Center III level times, to the churches seeking for prayers from the pasters, and the traditional healers. I moved to one clinic where they told me that my child was very pall and they referred me to the main hospital, Mbale. When I reached the main hospital they refused to see me because I had no mask. I had to go and look for a mask and this is when they attended to my child. The doctor who reviewed my child immediately ordered for blood and they transfused my child.

Jalia: If COVID-19 had not happened where would you seeking health care?

J**oan**:.Aaaa I have no particular health facility that I think I prefer, so long as they give me the treatment. I always look for a chip health facility. There is a health facility near here.

**Jalia:** How has this changed from before?

**Joan:**: I do not think there has been a change, only that my husband has no money now, that is the only problem, I am also worried about my child. There is a change after my child was transferred to the main hospital, the child is not responding to the treatment. I think those nurses were cheating me I discovered that they pretended to be treating my child yet, they were doing nothing. But I had no option that time I could not easily work to the main hospital.

**Jalia:** Who has initiated the changes?

There is no change only that now they have sent me to the main hospital. It is my husband who takes care of this child whenever it falls sick. I cannot answer some of these questions may be ask my husband, should I call him.

Jalia: No, we have already talked to him we want to hear from you.

**Joan:** …. You know he is the one who makes the decisions here. To me, I just follow what he tells me to do.

**Jalia:** What impact do you feel these changes have had on your care and that of your infant?

**Joan:** My child was going to die because I was reluctant to go to the main hospital because of covid pandemic, remember my husband had to go and obtain the permission to move using a motorcycle during the late ours.

**Jalia:** How long did take you to change

**Joan:** We spent about four months before we moved the main hospital. But this time my child was not okay he could not even eat, God is really with us. I used to go to the health centre III they prescribed drugs which I never even bought.

Jalia: What fears/ concerns do you now have?

**Joan:** The situation is really bad. I fear my child might die because the health workers are not vigilant though the child has improved I fear my child might die because at times I go to hospital and they tell me to pay for the blood.

**Joan:** The child has sickle cell disease the health workers at times ask for money. I cannot tell, … may be they are not well remunerated during this time of covid. I tell you I used to buy blood three times a week until my child improved. I used to visit the health facility, they prescribed drugs but I had no money to buy them most times we gave this child herbs. Look at that plant, that plant with red leaves, I used to boil it and give it to my child, it is very good for people with little blood (anaemia).

Jalia: Do you feel confident about the care provider you received?

**Joan:** Maybe I can say that I only trust the health workers at the main referral hospital. The care was good because I paid for it, so long as you pay, you gt the best treatment. I am happy with the treatment given to my child

**Jalia:** Do you think any other measures could have been taken to help you?

**Joan:** They should pay the health workers so that they stop taking money from us. They should ensure that there is enough blood for the patients’ kike my son I almost lost my child

**Jalia:** Did you/do you receive advice/care from any informal carers? If so, who?

**Joan:** No no health worker talked to me about the care to my child, but someone told me that the child was going to die why am I bothering myself

**Jalia:** Are you happy that your baby is healthy (whether born or not)?

**Joan:** I am now happy with my child I know he will survive.

**Jalia :** Thank you for participating in this study>
