# Supplementary material for: Experiences of mothers and significant others in accessing comprehensive healthcare in the first 1000 days of life post-conception during COVID-19 in rural Uganda
Source: BMC Pregnancy Childbirth. 2022 Dec 15;22:938. doi: 10.1186/s12884-022-05212-x (PMC9754309; doi:10.1186/s12884-022-05212-x)
Supplement: Supplementary file 6 — Additional file 6. [file 12884_2022_5212_MOESM6_ESM.docx]

.    **Interview Guide for the Women and their significant others**

**Title of the Study:** Experiences of social isolation and social distancing for women and the significant others in the family on continuity of care in the first 1000 days of life during the COVID 19 pandemic at Bunghokho-Motto Sub- County Mbale.

**Personal information**

**Anonymised Name**: Judi

Tell me more about yourself.

1. **Work:** Peasant
2. **Genda:** Male
3. **Age:** 67years
4. **Address**: Makere
5. **Marital status**: Divorced
6. Family: 11 children to father-in-law and 2 children to the woman
7. **Relationship**: Father-in-Law
8. **Education background**: No formal education
9. **Youngest child**: 3 months

**Interviewer G:**

2. What has been your experience of being cared for/care to a pregnant woman, labouring, postnatal, or infant during the time of the pandemic?

**Judi:**  This is my daughter-in-law; my son went to Kampala to work and left this girl in my care. He normally comes back home at least once a month. This time when the covid pandemic struck, he was not around. he was in Kampala. He left this woman pregnant, and she delivered 3 months back. I have been the one taking care of this woman, you see I am old, my ability to provide as I used to do is diminishing. During her pregnancy, I sent her to the hospital to see the doctors once, that is what I remember. But at times she would move away from home only to come back and tell me that she had gone to the hospital without telling me. At one point I thought she was looking for other men. Anyway, I cannot control her movements. At delivery she started laboring at night, this time at least we could move on a motorcycle in the morning. I contacted someone to come and take her to the hospital, I could not escort her because she has another young child hear at home.

**Interviewer G:** How old was the child then?

**Judi:** The youngest was I year, and the eldest was 3 years. You see this situation prevented me from escorting this woman to the hospital, so she went alone, I knew the health workers were there and they would take good care of her.

**Interviewer G:** If COVID-19 had not happened where would your pregnant woman, be seeking health care?

**Judi:** She delivered the previous babies from that health facility, HCIV. I think this is where I would have taken her.

**Interviewer G**: How far is this health facility from here?

**Judi:** About 10 miles I think, I used to walk that distance, but these lazy people nowadays tell you that this is far.

**Interviewer G**: How has this changed from before?

**Judi:** My dear friend, this time I had to tell her to go to the health centre near here. I know it is not as good as the other one but due to the challenges in transport and obtaining permission to move she had to go to the nearby health facility. But this they did a good job, I have not had any complaint from her.

**Interviewer G:** How far is the health facility from here?

**Judi:** I think it is 3 miles because I see this woman at times walking to the facility. She is a strong woman

**Interviewer G:** How has the care changed since she moved to this health facility?

**Judi:** I have never escorted her to the facility, therefore I do not know what kind of care she gets in this facility. She only communicates to me issues related to buying drugs. I think the care is good.

**Interviewer G:** Who makes decisions regarding the care of the daughter-in-law?

**Judi:** I make the decisions because my son is not here. The good thing he sends me some money, that is what I use to buy drugs and take care of her. I have not told you, these children at times fall sick. What I do I buy some drugs and give them and indeed they improve.

**Interviewer G:** Why don’t you take them to the hospital, why do you have to buy drugs?

**Judi:** There is covid in the health facilities, what will I tell my son when he comes back and finds them dead. I cannot risk it; I know the health workers in the drug shops I can even call them to come. I just describe to them the illness and give them the drugs.

**Interviewer G:** What impact do you feel these changes have had on your care/ on the care to a pregnant woman, laboring, postnatal, or infant?

**Judi:** To me, I have no problem one time this woman decided to take my grandson to the hospital without telling me. She only told me when she had come back and she wanted some money to buy the drugs. I felt bad because I had warned her never to move to the hospital with my grandchildren. I am the one who carries these children when this woman moves away from home, now image what if they contract the disease and they pass it on to me at this age, I will die.

**Interviewer G:** What fears/ concerns do you now have?

**Judi:** I have a feeling that this daughter-in-law might contract covid 19 from the health facilities and bring it here because she keeps on telling me that she is going to the health facility. The other time she took the baby, image to the hospital for immunization. Can we wait until covid has subsided then we take the children for immunization? For this young one, I refused her. Let the father come back from Kampala he will take her.

**Interviewer G:** Thank you for interacting with us.
