# Supplementary material for: Experiences of mothers and significant others in accessing comprehensive healthcare in the first 1000 days of life post-conception during COVID-19 in rural Uganda
Source: BMC Pregnancy Childbirth. 2022 Dec 15;22:938. doi: 10.1186/s12884-022-05212-x (PMC9754309; doi:10.1186/s12884-022-05212-x)
Supplement: Supplementary file 7 — Additional file 7. [file 12884_2022_5212_MOESM7_ESM.docx]

**Interview Guide for the Women and their significant others**

**Title of the Study:**

Experiences of social isolation and social distancing for women and the significant others in the family on continuity of care in the first 1000 days of life during the COVID 19 pandemic at Bunghokho-Motto Sub- County Mbale.

**Personal information**

**Anonymised Name**: Justine

Tell me more about yourself.

1. **Work**: Housewife
2. **Age**: 25 years
3. **Address**: Bukasakye
4. **Marital status**: Married
5. **Address:** Bukasakye
6. **Family:** 2 Children
7. **Youngest:** 6 months
8. **Education background**: Primary 3

**Interviewer G:** What has been your experience of being cared for/care to a pregnant woman, laboring, postnatal, or infant during the time of the pandemic?

**Justine:** I delivered during the time of the covid 19 pandemics. During pregnancy, I could go to the clinics to buy drugs from the clinics in the village, because it was not easy for me to access the health facilities. These drugs were not cheap; I remember I used a minimum of ten thousand (10,000/=) each time I decided to go to buy them.

**Interviewer G:** How did you decide on the drugs to buy?

**Justine:** You know I carried the treatment sheet with me, I obtained this treatment sheet from the main hospital Mbale. I tell you whenever I shallowed these drugs I felt better, I want to say that they worked. To me, this was a way to save. You imagine, at the health facility booking for antenatal was very expensive, there was that initial payment, I do not remember how much, my husband paid. Then each time I would pay 1,1000/=

**Interviewer G:** If COVID-19 had not happened where would you/ pregnant woman, laboring, postnatal, or infant in your family be seeking health care?

**Justine:** I used to go to the main hospital Mbale.

**Interviewer G:** How has this changed from before?

**Justine:**  I used to go to Mbale's main hospital, but when the covid pandemic struck us I had to go to the private clinic for antenatal. It was had to go to the Government hospitals because of the congestions and the long lines before you are attended.

**Interviewer G:**

Who has initiated the changes?

**Justine:** We sat; my husband and I and we agreed that l would buy drugs from the clinics. I continued swallowing the drugs that were prescribed from the main hospital. The nearby health facility has many mothers at one time I went there they did not exam me and they prescribed drugs for me. I remember I did not but those drugs, but I recovered from the fever.

**Interviewer G:** What impact do you feel these changes have had on your care/ on the care to a pregnant woman, laboring, postnatal, or infant?

**Justine:** During my first pregnancy, I was examined by the midwives at the facility, you know they used to talk to us and advise us on what to eat, but this time I missed out on this. I used to go to the antenatal clinic, interact with other pregnant women and we could sit and talk Maybe regarding treatment, I do not think I missed out a lot because I had the previous treatment forms, I would use them to buy drugs.

**Interviewer G:** What fears/ concerns do you now have?

**Justine:** I have a feeling that the health workers at one moment will stop working because of fear for covid.

**Grace Interviewer G:**  Do you feel confident about the care provider you received?

**Justine:** I tell you …. the health workers at the HCIII have some issues, you cannot give me drugs without examining me. I see there is a problem there. Maybe the midwives were fearing contracting the coronavirus.

**Interviewer G:** Do you think any other measures could have been taken to help you?

**Justine:** The government should build another hospital and equip it with the necessary equipment and nurses and midwives.

**Interviewer G:** Did you/do you receive advice/care from any informal carers? If so, who?

**Justine:** I did not receive advice from any other person.

**Interviewer G:** Are you happy that your baby is healthy (whether born or not)? If no, are you planning to seek other help? From whom?

**Justine:** My baby is missing some benefits like milk which we cannot afford to buy now. I am somehow confused about the type of food to give my baby. Anyway, what I will do I will mix been soup and matooke (plantains) and give, I have nothing to do.

**Interviewer G:** Thank you for participating in the interview.
