# Supplementary material for: Experiences of mothers and significant others in accessing comprehensive healthcare in the first 1000 days of life post-conception during COVID-19 in rural Uganda
Source: BMC Pregnancy Childbirth. 2022 Dec 15;22:938. doi: 10.1186/s12884-022-05212-x (PMC9754309; doi:10.1186/s12884-022-05212-x)
Supplement: Supplementary file 8 — Additional file 8. [file 12884_2022_5212_MOESM8_ESM.docx]

## .      **Interview Guide for the Women and their significant others**

**Title of the Study:**

Experiences of social isolation and social distancing for women and the significant others in the family on continuity of care in the first 1000 days of life during the COVID 19 pandemic at Bunghokho-Motto Sub- County Mbale.

**Personal information**

**Anonymised Identifier:** Kauda

Tell me more about yourself.

1. **Work**: Farmer
2. **Age:** 32 years
3. **Marital status**: Single
4. **Address**: Makere
5. Family: 6 children
6. Youngest child; 5 months
7. Education background: P.5

**Interviewer G:**  What has been your experience of being cared for/care to a pregnant woman, labouring, postnatal, or infant during the time of the pandemic?

**Kauda:** I have had a tough experience, the lockdown found me pregnant I could not work because there was no transport to go to Mbale hospital and the pregnancy was big yet, I had to take my monthly drugs for HIV. I always pick the drugs monthly. Similarly, I had to attend antenatal clinics. So the challenge I had I could not go to pick my HIV drugs and at the same time to attend antenatal clinic.

**Kauda:** When I came to the time of delivery, I was not able to go to hospital I delivered from home. I bled a lot I was in a bad state. Days after my feet and legs become swollen. We had no money and the feeding was not the best. I stayed here with my mother. During the time I was attending antenatal clinic they told me that if my baby takes a syrup immediately after birth it will not get the HIV. I was advised to deliver from the health facility. My baby did not get this syrup. There was no transport to the health facility

**Interviewer G:** Did the previous baby take the syrup?

**Kauda:** No he did not

**Interviewer G:** Is this baby free from HIV?

**Kauda:** We stayed here at home in a confused state. We had no money, I did not get treatment. When the situation improved I travelled to the health facility to pick my drugs and the syrup for the baby because, they said when I deliver immediately I had to go and pick the syrup for the baby My child missed out on this chance I am worried.

**Interviewer G**: Have they screened this baby?

Kauda. Yes, he was ok.

**Interviewer** G: What were the results?

**Kauda:** The baby is fine, they told me to go back when the child makes 6 months.

**Interviewer G**: How old is the baby?

**Kauda**: He is 5 months old now

**Interviewer G**: Is the baby still fine now?

**Kauda:** Yes, he has no problem, they told me I have to breastfeed the baby, I should not give any other feeds.

**Interviewer G**: Have you done so?

**Kauda:** Yes, I only breast feed, the covid time did not treat me well.

**Interviewer G**: Tell me more about your delivery experience.

**Kauda:** I bled a lot, I almost died they told me that I had retained materials after birth. What my mother did was to contact a nurse who came and massaged my abdomen. She tried to remove the blood using her hands; this was very painful.

**Interviewer G:** Was your baby Immunised?

**Kauda:** Yes, after two weeks I took the baby for immunisation, I could not work before then.

**Interviewer G:** Have you taken this child back to Hospital?

Kauda: Yes, but the child was taken back because of the cord, it was my mother who went to hospital with the baby me, I could not walk. They found out the cloth that was used to tie the cord was full of blood. I tell you me and my baby almost died.

**Interviewer G**: That was bad. If COVID-19 had not happened where would be seeking health care?

**Kauda:** I would have gone to the main hospital.

**Interviewer G:** How has this changed from before?

**Kauda:** I delivered from home because there was no transport, the motorcyclists refused to take me to hospital, so I remained at home during the time of labor.

**Interview G:** Who made this decision?

Participant: It was my mother who said that I can deliver from home. She would manage.

**Interviewer G:** What impact do you feel these changes have had on your care or the baby?

**Kauda:** I have no job; my children are all here with me I have no support from their fathers.

**Interviewer G**: Are the fathers different

**Kauda:** Yes, two are from the same farther and this young one is from another man. My children are about to back to school, but I have nothing to give them. I am supposed to take in a lot of fluids, but I have nothing to drink and eat. Remember I am supposed to breastfeed without other feeds. I had a difficult time, my legs and feet all became swollen because I had no blood. I have nothing my mother has been preparing for me greens, covid pandemic really mistreated me.

**Interviewer G:** What fears do you have?

**Kauda:** J am worried about my baby, I delivered from home, yet I was supposed to deliver from the hospital, he did not get the syrup that prevents HIV to the baby.

**Interviewer G:** Are you confident with the care givers?

**Kauda:** That is what it is.

**Interviewer G**: Elaborate on this

**Kauda:** You may go to see the health workers, but the health worker takes long to see you. These health workers distanced themselves from us. I once had something to tell them, but I could not tell them because I sat very far from the health worker who attended to me, she never even examined me. She asked me questions when everyone was hearing. I just told her that I had a headache, yet I had another issue. They gave me treatment without examining me. These health workers were very few they had nothing to do. The other thing I think about is when you go to hospital for immunization without a mask, they do not attend to you. They chess you with the baby. The first time I took the baby to hospital, I had to come back home without the baby being seen. So I had to go back the following day.

**Interviewer G:** Have you received advise from another person?

Kauda: No but I have some fears

**Interviewer G**: What fears do you have?

Kauda: I feel that the days for going back for review are long, you need to wait until the set date comes for you to seek health care. These days I use health center three, the other facilities are private.

**Interviewer G:** Thank you for giving me this information:

.
