# Supplementary material for: Experiences of mothers and significant others in accessing comprehensive healthcare in the first 1000 days of life post-conception during COVID-19 in rural Uganda
Source: BMC Pregnancy Childbirth. 2022 Dec 15;22:938. doi: 10.1186/s12884-022-05212-x (PMC9754309; doi:10.1186/s12884-022-05212-x)
Supplement: Supplementary file 9 — Additional file 9. [file 12884_2022_5212_MOESM9_ESM.docx]

**Interview Guide for the Women and their significant others**

**Title of the Study:** Experiences of social isolation and social distancing for women and the significant others in the family on continuity of care in the first 1000 days of life during the COVID 19 pandemic at Bunghokho-Motto Sub-county Mbale.

**Anonymised Name:** Margaret

Tell me more about yourself.

1. **Work**: Housewife
2. **Age:** 30 years
3. **Gender:** Female
4. **Address**: Makere
5. **Marital status**: Married
6. **Family**: 7 children
7. **Youngest child** 4 months
8. **Education background**: Senior 4

**Interviewer G:** What has been your experience of being cared for/care to a pregnant woman, laboring, postnatal, or infant during the time of the pandemic?

**Margaret:** It was difficult to access care during this time. I missed out on going for antenatal. Because there was no transport.

**Interviewer G:** How many times did you visit the health facility?

**Margaret:** I went there three times only.

**Interviewer G:** How many times do you always attend antenatal care?

**Margaret:** About 4 or 5 depending on my health status. The time when was feeling sickly, I would even go for Antenatal 7 times. This time I missed out.

**Interviewer G:** If COVID-19 had not happened where would you/ pregnant woman, laboring, postnatal, or infant in your family be seeking health care? **Margaret:** **Participant:** My husband would have taken me to the main hospital.

**Interviewer G**: Who has initiated the changes?

**Margaret:** It was my husband.

**Interviewer G**: How has this changed from before?

**Margaret:** I started laboring during the daytime but we could not access the permission late easily and transport to the hospital was not readily available. Therefore, I was taken to the hospital at night, When we reached the health facility, the health workers could not help me because they were busy and very few as compared to the number of mothers who were laboring. I remained in bed until one midwife came and examined me. She told me that I was not going to deliver normally, I was going for a Caesarean section. She consents me and I had to wait for the doctor to come. During this time as I was waiting, I got a strong contraction and in a few minutes I push the baby out. They ran and picked the baby from the bed.

**Interviewer G**: How did the baby behave immediately after birth?

**Margaret:** One midwife run away with my baby and I even got worried. Later on, they brought the baby back and requested me to breastfeed it. I could not breastfeed the baby because I was very weak.

**Interviewer G**: What else did they tell you about the baby?

**Margret:** They told me that they were helping the baby to breathe. This baby never cried.

**Interviewer G**: What happened thereafter.?

**Margaret:** I bleed a lot. I had them talking about transfusing me but there was no blood. They brought back my baby and they told me to breastfeed. This time I was feeling a lot of dizziness. I could not walk. I stayed at the health facility for two days until they discharged me home.

**Interviewer G:** What impact do you feel these changes have had on your care/ on the care to a pregnant woman, laboring, postnatal, or infant?

**Margaret:** This time I almost died. There were so many delays. I think that is why I bled. The bleeding stopped after one month.

**Interviewer G**:: Do you feel confident about the care provider you received?

**Margaret:** Yes I think they are all competent, but none of them came near me, yet I had issues that I wanted to tell the midwife without any other person hears.

**Interviewer G:** Did you receive advice/care from any informal carers? If so, who?

**Margaret::** No I did not

**Interviewer G:** Are happy that your baby is healthy (whether born or not)? If no are you planning to seek other help? From whom?

**Margaret:** The baby has remained weak. The last time I took her for immunization they told me that she was small compared to the age.

Did the health workers tell you what to do?

**Margaret:** They told me to continue breastfeeding but I could not give only the breast milk because at times I feel that there is nothing the baby is sackling. So add some bananas and soup.

**Interviewer G:** Thank you for interacting with me.

.
