# Supplementary material for: Experiences of mothers and significant others in accessing comprehensive healthcare in the first 1000 days of life post-conception during COVID-19 in rural Uganda
Source: BMC Pregnancy Childbirth. 2022 Dec 15;22:938. doi: 10.1186/s12884-022-05212-x (PMC9754309; doi:10.1186/s12884-022-05212-x)
Supplement: Supplementary file 10 — Additional file 10. [file 12884_2022_5212_MOESM10_ESM.docx]

## **Interview Guide for the Women and their significant others**

**Anonymised Identifier**: Mike

**Title of the Study:**

Experiences of social isolation and social distancing for women and the significant others in the family on continuity of care in the first 1000 days of life during the COVID 19 pandemic at Bunghokho-Motto Sub-county Mbale.

  Personal**information**

Identification Number: Participant 25

Tell me more about yourself.

1. **Work:** Self Employed
2. **Age:** 30
3. **Marital status**: Married
4. **Address**: Bukasakye
5. **Family**: 2 Children
6. **Youngest**: 6 months
7. Education background: Primary 7

**Interviewer G:** What has been your experience of being cared for/care to a pregnant woman, laboring, postnatal, or infant during the time of the pandemic?

**Mike:** My wife was delivered from the main hospital Mbale. I March. When the Covid pandemic struck my child had only received one dose of Immunisations. There was no transport, I decided that she should not go to Mbale because there was a risk of contracting Covid 17 virus. I decided that we wait until June, wh went to Health Center III. The child was given all the immunization doses that he had missed, at the same time. When we reached how that day, we had a terrible experience after the immunization, the child cried and we did not sleep for two days. I started thinking that maybe I will never take back my child for immunization. Later on, In July, I changed my mind to help my chid and take it back to complete the immunization schedule. He has now completed the dose at six months.

I used to work in the industrial ara but at least I dad food I sold all my goats and chicken to support my family during the difficult time of covid, I then God I succeeded.

**Interviewer G:** If COVID-19 had not happened where would you/ pregnant woman, laboring, postnatal, or infant in your family be seeking health care?

**Mike:** I am sorry to tell you this but the health workers at health center III are not good they demand money yet in the main hospital everything is free I could only look for transport money but here it is terrible they demand money, remember my wife gave a 10,000/= the health worker refused it, she said that treatment is free. But these health workers demand 70,000/= if you deliver a boy and 50,000/= when you deliver a girl. I asked myself, what is the difference between a boy and a girl, they are all delivered the same way. That is why I prefer the main hospital Mbale. The only fear I had that time was that if police found you on the road without a permission letter to move, that would be a crime, and you would risk being beaten up.

**Interviewer G:** How has this changed from before?

**Mike:** My wife has been seen by the nurses at the health center III I am not sure whether the treatment they offer is appropriate. But again another fear that I have at the main hospital they are very strict with people wearing masks. Could it be that there are many people with covid 19 virus at this health facility? Here in these facilities around us, it is not a must. It was so scary that at one point had to buy drugs instead of going to the hospital in fear of contracting the coronavirus. From the health facility

**Interviewer G:** Who has initiated the changes?

**Mike:**: Nobody initiated this change, the situation that prevailed predicted the decision to take. For example, one would not insist on going to a given health facility if the circumstances could not allow you to move you see…..

**Interviewer G:** What impact do you feel these changes have had on your care/ on the care to a pregnant woman, laboring, postnatal, or infant?

**Mike:** I have not had a big change in my family because I had enough money to provide food and support for my family during the time of covid. I sold many of my livestock. I bought drugs for my wife each time she was not feeling well. I decided that we limit the number of times she moved to the hospital in fear of contracting covid from the health facility. The other thing is my wife has had time to at least stay with me for some good time, something that has not happened before, but this comes with poverty.

**Interviewer G:** What do you think about this situation?

**Mike:** Let me tell you if a man has no money, even the social life fails I need money to become a complete man you know (laughed).

**Interviewer G:** What fears/ concerns do you now have?

**Mike:** The fear that I have is that time will come when we shall lose our families; the families are going to separate because if a child falls sick and you can not provide as a man or take the child to the hospital, then what is your use at home. There are many other issues in the home related to stress. I can not mention all. In some instances, my wife keeps quiet without talking and I ask myself what have I done to this woman. But I think all this is due to my poverty now.

Interviewer G: Mike thank you for sharing.
