# Supplementary material for: Experiences of mothers and significant others in accessing comprehensive healthcare in the first 1000 days of life post-conception during COVID-19 in rural Uganda
Source: BMC Pregnancy Childbirth. 2022 Dec 15;22:938. doi: 10.1186/s12884-022-05212-x (PMC9754309; doi:10.1186/s12884-022-05212-x)
Supplement: Supplementary file 11 — Additional file 11. [file 12884_2022_5212_MOESM11_ESM.docx]

.  **Interview Guide for the Women and their significant others**

**Title of the Study:**

Experiences of social isolation and social distancing for women and the significant others in the family on continuity of care in the first 1000 days of life during the COVID 19 pandemic at Bunghokho-Motto Sub- County Mbale.

**Anonymised Identifier: Rose**

Tell me more about yourself.

1. **Age**: 24 Years
2. **Gender**: Female
3. **Marital status:** Married
4. **Address:** Bukasakye
5. **Family:** 2 Children
6. **Youngest child**: 18 months
7. **Work:** Housewife
8. **Pregnancy**: 9 months
9. **Education background**: P5

**Interviewer G:** What has been your experience of being cared for/care to a pregnant woman, labouring, postnatal, or infant during the time of the pandemic?

**Rose:** Accessing health care has been a problem, but my husband has tried his level best to ensure that I receive treatment from the health facility. At the health facility, I was given a mosquito net. There is an organization at HCIV when one pays four thousand shillings (4000/=) you get a voucher card that one uses to get everything that is needed during pregnancy and delivery. I think that I have been taken care of well. The problem I had during covid I did not start early, I started with the HCIII, where these services are missing. But all the same, I can now get the services.

**Interview G:** Your husband said that during the covid time your child fell sick, what was your experience?

Rose: I have remembered, this was not easy, the child was sick, it refused to eat and yet it was vomiting and having diarrhoea. The child was very weak, by the time we reached the hospital the child was almost dying. But God was on our side. The health workers worked hard, I thank them.

**Interviewer G:** If COVID-19 had not happened where would you/ pregnant woman, labouring, postnatal, or infant in your family be seeking health care?

**Rose**: I used to go to the HCIV I had a voucher I had all the treatment that was ordered but when I moved to HCIII, the story changed. I could sit from morning to evening waiting for treatment. This was due to the big number of mothers at the health facility and yet the health workers were few.

**Interviewer G:** How has this changed from before?

**Rose:** The health workers are few and they take time seeing one mother Second they make you sit far from their working table. I know they feared contracting covid the distancing was too much. That time, I sat at the facility for so long. I had gone to tell them that I was vomiting so much every morning I just wanted their advice but I moved and went back home without treatment.

**Interviewer G:** How did you manage this problem?

**Rose:** I went and just bought drugs from the pharmacy.

**Interviewer G:** Who made this decision?

**Rose:** I just decided to go because it was too much for me.

**Interviewer G:** What impact do you feel these changes have had on your care/ on the care to a pregnant woman, labouring, postnatal, or infant?

**Rose**: There has been a lot of time-wasting at the health facility, I used to leave my child alone at home, to go to the health facility, I would come back and find her unattended, this was so risky. I used to move with the young one.

**Interviewer G**: How old is this child?

**Rose:** The eldest is 4 years

**Interviewer G**: Do you feel confident about the care provider you received?

**Rose:** Yes, the health workers know what they are doing, they are just overwhelmed with the numbers and the fear of contracting covid.

**Interviewer G:** Do you think any other measures could have been taken to help you?

**Rose:** I do not think so

**Interviewer G:** Did you/do you receive advice/care from any informal carers? If so, who?

**Rose**: No, I have never received any advice from any other person apart from the health workers. Maybe those who sell the drugs. But they are also health workers.

**Interviewer G:** Are you happy that your baby is healthy (whether born or not)? If no, are you planning to seek other help? From whom?

**Rose:** My baby used to fall sick that time when I had just conceived but now it is fine, no problem

**Interviewer G:** Rose thank you for participating in the interview.

.
