# Supplementary material for: Experiences of mothers and significant others in accessing comprehensive healthcare in the first 1000 days of life post-conception during COVID-19 in rural Uganda
Source: BMC Pregnancy Childbirth. 2022 Dec 15;22:938. doi: 10.1186/s12884-022-05212-x (PMC9754309; doi:10.1186/s12884-022-05212-x)
Supplement: Supplementary file 12 — Additional file 12. [file 12884_2022_5212_MOESM12_ESM.docx]

**Interview Guide for the Women and their significant others**

**Title of the Study:**

Experiences of social isolation and social distancing for women and the significant others in the family on continuity of care in the first 1000 days of life during the COVID 19 pandemic at Bunghokho-Motto Sub-county Mbale.

**Anonymised Identifier: Sam**

Tell me more about yourself Sam.

1. **Work**: Boda Boda Rider
2. **Age:** 35
3. **Male**
4. **Address:** Luyehe B
5. **Marital status**: Married
6. **Family:** 4 Children
7. **Youngest** child: 3 Months
8. **Education background**: P.5

**Interviewer G:** What has been your experience of being cared for/care to a pregnant woman, laboring, postnatal, or infant during the time of the pandemic?

**Sam:** During covid, I had a challenge, I walked with my wife to the hospital. She used to come back with swollen feet. I used to get a piece of cloth deep in water and massage her feet. I feared that they would burst at one time because they were swollen. The nurses at this facility were very good, they always attended to her first the moment we reached the health facility. The problem I had was that I saw my wife safer from hunger because I had no money to buy food. I used to borrow a motorcycle from my friend to go and make some money one day as I was riding back home I got into an accident. I was taken to the hospital with a broken bone (Fracture) below the knee. I moved on cratches for 2 months with the leg In a plaster. During this time my friend helped me to take my wife to the hospital to deliver She had to stay in the hospital for 3 days because the wife had a small baby. Later on, she was discharged. This bay has been a crying baby even when the mother puts her on the breast. He has remained small and weak. Anyway, I have now recovered completely, I can move and take care of my family. ( went silent).

**Interviewer G**: Is there anything you want to add to your experience?

**Sam**: Yes… We settled In this place from Bududa, a place of landslides. I have no relatives around here. My children have been falling sick one after the other. During covid time, you see that one, he is now two years, but I used to carry him on my back to the health facility,

**Interviewer G**: How fur is a health facility?

**Sam**: It is 3 miles from here. This is the nearest the nurses at this health facility are good only that they are few.

**Interviewer G**: If COVID-19 had not happened where would you/ pregnant woman, laboring, postnatal, or infant in your family be seeking health care?

**Sam**: I always take my wife to the bigger hospitals

**Interviewer G**: How has this changed from before?

**Sam:** This time it was not possible due to the covid pandemic she delivered from Health Center III. This is not what I wanted

**Interviewer G:** Who has initiated the changes?

**Sam**: I made this decision

**Interviewer G:** What impact do you feel these changes have had on your care/ on the care to a pregnant woman, laboring, postnatal, or infant?

**Sam:**  My baby has been folling sick since birth maybe this is the reason why. The other thing is before the covid pandemic I used to ride my motorcycle and earn some money but now since my motocycle broke down during the covid 19 pandemic I have not to work apart from borrowing from my friends. The other issue is that if a child falls sick and you go to the health facility the nurses are few I reached early in the morning one day at the health facility but I left this place late in the evening. The nurses came late and they left early. Second, I used to move with this woman to the hospital each time I took the children to the hospital, she would come back home very tired very moody and annoyed as if I am the one who told the health workers to delay the services. Remember to me I would carry the baby, but she would remain in this state of anoyence for days, this would work me up. I had a painful leg, let me tell you we were psychologically affected.

**Interviewer G:** What fears/ concerns do you now have?

**Sam** : If this situation continues the families are going to break because of the psychological issues, created by poverty and so many different issues. The health workers fear us thinking that we have covid and we are going to infect them. I am sure this feeling also affects the care they provide the patients. This is my feeling, do not miss understand me.

**Interviewere G:** Do not worry we just want to know what your thoughts are. Thank you for participating in this discussion.
