# Supplementary material for: Experiences of mothers and significant others in accessing comprehensive healthcare in the first 1000 days of life post-conception during COVID-19 in rural Uganda
Source: BMC Pregnancy Childbirth. 2022 Dec 15;22:938. doi: 10.1186/s12884-022-05212-x (PMC9754309; doi:10.1186/s12884-022-05212-x)
Supplement: Supplementary file 13 — Additional file 13. [file 12884_2022_5212_MOESM13_ESM.docx]

**Interview Guide for the Women and their significant others**

**Title of the Study:**

Experiences of social isolation and social distancing for women and the significant others in the family on continuity of care in the first 1000 days of life during the COVID 19 pandemic at Bunghokho-Motto Sub- County Mbale

**Personal information**

**Anonymised Identifier:** Sonia

Tell me more about yourself.

1. **Work**: Housewife
2. **Age:** 24 years
3. **Marital status:** Married
4. **Address:** Makere
5. **Family**: 3 children
6. **Education background**: P.7

**Interviewer G**

2. What has been your experience of being cared for/care to a pregnant woman, labouring, postnatal, or infant during the time of the pandemic?

**Sonia:** During the time of the covid pandemic I walked to the health facility alone. On reaching the health facility, I would find a long line of mothers waiting to be seen by the health workers. I remember, at least twice I went back home without being seen, and yet I was not feeling well.

**Interviewer G:** What happened that time?

**Sonia:** The health workers told us that it was too late, they had to stop and go home as they feared that they would be caught up by the curfew time before reaching home. This was understandable, but remember I had to walk back home about 3 km, that was not so easy.

What happened at the time of delivery?

**Sonia:** My father-in-law called a motorist who transported me to the health facility, still I went along as my husband works in Kampala. But he left me in the hands of my father-in-law. He told me that he had to stay and take care of the young ones. I reached the health facility at 9.00 am and I delivered at around 6.00 pm. This has been the longest I have stayed in labor. I always reach the health facility and delivery and after a few hours, I would go home. This time I had to sleep at the health facility and I moved home the following day.

**Interviewer G:**: Who assisted you at the health facility?

**Sonia:** You know the health workers knew me because I used to give them some little money after taking care of me during antenatal, therefore, they supported me. Similarly, I got support from the attendants to the women who had come to deliver. The fear I had was that they could still my baby therefore, I did not leave my baby with any of them at any time. I remember I did not bathe until I reached home. I was discharged in the morning. I had a small bag, I carried it with my baby and I reached home. It was so funny, my father-in-law who never called me to find out what I was going through when I reached home was the happiest person to see the baby. Anyway, those are our men here.

**Interviewer G:** If COVID-19 had not happened where would you/ pregnant woman, laboring, postnatal, or infant in your family be seeking health care?

**Sonia:** I would have gone to the health center IV for the antenatal visits. The place is far from here, so during the time of covid, I could not go there. Though their care is better. Then I would have delivered from The Traditional Birth Attendant near here.

**Interviewer G:** How has this changed from before?

**Sonia:** My husband was not around to escort me because he was held up in Kampala. I have always gone for antenatal at the health facility, but deliver with the TBA. When I visited my husband in Kampala around January this year, I went to one of the health facilities in Kampala. They told me that I should never go to a traditional birth to attend again. Therefore, this time I decided to go to the hospital. Similarly, this time, my child was immunized immediately after birth. I have always gone home without the baby being immunized, I have always done it after one week.

**Grace**: Were you able to go back for immunization after the initial dose?

**Interviewer G:** Yes, but it was hard because I had to walk to the health facility as my father-in-law never wanted me to take the child to the hospital in fear that the child would get covid 19. What I did, I moved out of the house without his knowledge and I took the baby to the hospital. Let me tell you, even that child used to fall sick, I would just escape and take the child to the hospital, then come to ask for money to buy the drugs.

**Interviewer G:** Who has initiated the changes?

**Sonia:** I have learned how to decide on my own because at times the decisions my father-in-law takes are not appropriate. But I never argue with him because he is a hot-tempered man.

**Interviewer G:** What impact do you feel these changes have had on your care/ on the care to a pregnant woman, laboring, postnatal, or infant?

**Sonia:** As I have already told you, I stay with my father-in-law, and he is the one who decides where I have to go for treatment, though this time I refused to go to the TBA. When the children fall sick he looks for herbs and treats them, in most cases, they get better. Many times I have walked to the health facilities without his knowledge. At the health facility they always prescribed drugs, but some of the drugs are not available in their pharmacy. This is when I come to him to request money to buy the drugs. I tell you, life has not been so easy for me.

**Interviewer G:** What fears/ concerns do you now have?

I have no fears because my baby has been taken care of well.

**Specifically, to the women**

**Interviewer G:** Do you feel confident about the care provider you received?

**Sonia:** Indeed, I am confident about the care the health workers provide. They are very knowledgeable only they do not have all the drugs we need.

**Interviewer G**

1. Do you think any other measures could have been taken to help you?

**Sonia:** No I cannot talk about the health workers; they are doing their best.

Grace

1. Did you/do you receive advice/care from any informal carers? If so, who?

**Participant 26B:** No I have not received any advice from other people, apart from the health workers

**Interviewer G:** Are you happy that your baby is healthy (whether born or not)? If no are you planning to seek other help? From whom?

**Sonia:** My baby is doing well. The health workers in Kampala told me that after delivery my baby should be immunized immediately and I should breastfeed for……... I think six months. I took their advice; I will always take my children for immunization.

**Interviewer G:** That is good. Thank you for sharing.
